# Neurotensin-Based Radioligands for NTS_1_R-Targeted Cancer Theranostics: Background, Current Status and Future Trends

**DOI:** 10.3390/ph19101623

**Published:** 2026-10-13

**Authors:** Berthold A. Nock, Panagiotis Kanellopoulos, Theodosia Maina, Eric P. Krenning

**Affiliations:** 1 Molecular Radiopharmacy, INRaSTES, NCSR “Demokritos”, 15341 Athens, Greece; nock_berthold.a@hotmail.com (B.A.N.); kanelospan@gmail.com (P.K.); 2 Cyclotron Rotterdam BV, Erasmus MC, 3015 CE Rotterdam, The Netherlands; erickrenning@gmail.com

**Keywords:** neurotensin, neurotensin subtype 1 receptor, cancer theranostics, targeted radiopharmaceuticals

## Abstract

**Background/Objectives**: The neurotensin subtype-1 receptor (NTS_1_R) is overexpressed in a number of human tumors, representing a valid biomolecular target for neurotensin-based radionuclide carriers. The latter are designed to deposit diagnostic–therapeutic radionuclides specifically on cancer lesions. This review summarizes research efforts toward the development, preclinical evaluation and first clinical outcomes of neurotensin-based radioligands in the diagnosis and therapy—radiotheranostics—of NTS_1_R-positive cancer. **Methods**: The preclinical and few clinical studies related to NTS_1_R-targeted radiotheranostics are discussed. The crucial issue of in vivo metabolic stability is highlighted. **Results**: Much effort has been dedicated to the development of effective NTS_1_R-directed radioligands for cancer theranostics, most of which are peptide-based. However, clinical success has been sub-optimal thus far, partly as a result of their fast degradation in the biological milieu. **Conclusions**: The administration of key protease inhibitor regimens, the introduction of branched or otherwise stabilized analogs and the advent of non-peptidic NTS_1_R-antagonists are some of the new promising approaches towards achieving clinical breakthroughs in the battle against NTS_1_R-expressing cancer.

## 1. Introduction

The clinical success of radiolabeled somatostatin analogs in the diagnosis and therapy—radiotheranostics—of somatostatin receptor-positive neuroendocrine tumors (NETs) [1,2] has set the groundwork for developing peptide radioligands directed to other receptor-targets highly expressed on human tumors [3,4,5,6,7]. Receptors of native peptides are members of the superfamily of G-protein coupled receptors (GPCRs). They are located on the cell membrane of target cells and are involved in the regulation of many physiological processes in the body [8]. Most importantly, GPCRs are highly expressed in various types of human cancer [3,9,10] and may thus serve as biomolecular targets for those cancers. In this regard, native peptides can be smartly remodeled to carry clinically relevant radionuclides to their cancer-residing GPCR-targets. Clearly, the new peptide radioligands need to fulfil important criteria at the preclinical level before being considered for clinical evaluation, such as high receptor affinity, sufficient metabolic stability and favorable pharmacokinetics in animal models, that is, high tumor uptake and retention vs. fast clearance from the body via the urinary system [5].

The design of new analogs has been greatly supported by the availability of motifs originating from native peptides as well as from the rich bank of registered drugs, of which more than 50% target GPCRs [10]. Typically, a bifunctional chelator (BC) is attached at the N-terminus of a peptide analog, directly or via a linker or spacer, thus allowing for the stable binding of the desired radiometal (Figure 1) [11,12,13,14].

The latter may be a photon emitter (e.g., Tc-99m, In-111) for single photon emission computed tomography (SPECT) or a positron emitter (e.g., Ga-68, Cu-64, Co-55) for positron emission tomography (PET) diagnostic imaging. When a particle emitter is applied (e.g., Lu-177, Tb-161, Ac-225), radionuclide therapy is considered [15,16,17]. Imaging is crucial for the initial diagnosis, staging and estimation of disease spread. It will guide patient selection for individually tailored therapies. Eventually, it is important for monitoring therapeutic outcomes and the following-up of patients in the post-therapy period, thus perfectly harmonizing with the principles of contemporary precision medicine [18,19].

Along these lines, a good number of radiolabeled neurotensin (NT) analogs have been developed since the 1990s as theranostic candidates for human tumors expressing the neurotensin subtype 1 receptor (NTS_1_R). In this review we shall report on the development of theranostic NTS_1_R-directed peptide radioligands, predominantly those based on NT, from the early days to now. Our report will focus on analogs labeled with diagnostic or therapeutic radiometals, whilst F-18 analogs solely destined for PET imaging will not be discussed. For more information on radiofluorinated NT-based analogs for PET imaging, on NT–dye conjugates for optical imaging, or on an application-oriented overview on NTS_1_R-targeting modalities with clinical impact, the reader is directed elsewhere [20]. In the present report, emphasis is given to efforts implemented to address the challenge of the insufficient metabolic stability of NT-based peptide radioligands, eventually compromising clinical outcomes (Section 2 and Section 3). The most important approaches to tackle the problem of stability will be detailed, and include, except for the administration of key protease-inhibitor regimens, the development of branched or otherwise stabilized analogs and the recent advent of non-peptidic NTS_1_R-antagonists.

In view of the plethora of structural modifications attempted over the years toward higher stability and concurrently toward the improvement of the NTS_1_R-targeting efficacy of radiometal-carrying NT conjugates, this review encompasses, three major sections: (a) Section 4, reporting on analogs suitable for labeling with Tc-99m (and radio-Re), (b) Section 5, dedicated to analogs suitable for labeling with theranostic bi/trivalent radiometals, for SPECT, PET and radionuclide therapy, and (c) Section 6, dedicated to non-peptidic radiolabeled NTS_1_R-antagonists. Each of the above sections is divided in subsections according to similar chemical modifications operated on original lead structures. The impact of chemical structure changes on the stability of end molecules and consequently, on tumor-targeting efficacy and overall biodistribution patterns is critically commented and discussed according to a structure–activity relationship (SAR) perspective.

The first two Sections (Section 1 (Introduction) and Section 2 (Neurotensin and Neurotensin Receptors)) are dedicated to outlining the rationale underlying the concept of radiotheranostics as well as the impact of NTS_1_R-targeting in anti-cancer drug development, providing ample references for the interested reader. In Section 3 (Metabolic Stability Considerations) the involvement of key proteases in the rapid catabolism of NT and its analogs is specifically elaborated.

## 2. Neurotensin and Neurotensin Receptors

Neurotensin (NT, pyroGlu-Leu-Tyr-Glu-Asn-Lys-Pro-Arg-Arg-Pro-Tyr-Ile-Leu-OH) is a linear tridecapeptide first isolated from bovine hypothalamus extracts [21,22]. NT is implicated in several physiological functions in the brain and the gut, including the regulation of pain and body temperature, as well as food intake and body weight control [23]. NT exerts its various actions after binding to specific receptors, comprising three subtypes, NTS_1_R, NTS_2_R and NTS_3_R [24]. The first two belong to the superfamily of GPCRs displaying a characteristic seven-transmembrane serpentine-like structure, whilst NTS_3_R is a sortilin-like receptor with a single membrane-spanning chain [25]. While NTS_1_R, NTS_2_R and NTS_3_R are all found in the brain, the NTS_1_R is additionally expressed in the gut, especially in the small intestine mucosa [24,26]. The NTS_1_R binds NT with sub-nanomolar affinity (0.1–0.3 nM) and is non-responsive to levocabastine, a nonpeptide histamine H1 receptor antagonist. In this way, it can be pharmacologically distinguished from the NTS_2_R, which shows less binding affinity to NT (3–5 nM) and is sensitive to levocabastine [27].

Of particular interest is the role of NT and NTS_1_R in cancer onset and propagation [28,29,30,31,32,33,34,35,36,37,38,39]. Thus, the high expression of NTS_1_R has been documented in a multitude of human cancers, including colorectal carcinoma [31,36,39], pancreatic ductal adenocarcinoma (PDAC) [40,41,42], lung cancer [35], Ewing’s sarcoma [43], prostate cancer [44,45], and mammary carcinoma [46,47]. These findings provide the molecular basis for employing NT-based carriers to deliver medically useful radionuclides to cancer lesions for diagnosis and therapy, in line with the somatostatin paradigm [1,2]. It should be noted, however, that, unlike the applied metabolically robust somatostatin cyclic octapeptide analogs [48], NT is a linear peptide susceptible to rapid degradation by proteases (vide infra) [49,50]. Another significant difference is the lack of undesirable pharmacologic or proliferative effects induced by somatostatin analogs [48], as opposed to NT and its analogs. The latter are potent agonists [26,32], activating the NTS_1_R upon binding. Binding prompts the rapid internalization of the NT/NTS_1_R complex and triggers a cascade of downstream events in target cells, provoking both acute systemic pharmacological effects in the gastrointestinal tract (GIT) and mitogenic effects [28]. All the above biological responses need to be taken into serious consideration during the design of effective and biosafe NT-analogs in the battle against NTS_1_R-positive human cancer. It should be mentioned that the C-terminal NT(8–13) represents the essential peptide fragment preserving full interaction capabilities with the NTS_1_R [32,51]. Hence, most radiolabeled NT analogs developed thus far as anti-cancer radiotheranostic candidates have been based on the NT(8–13) motif [52,53].

The intense search for clinically successful radiotheranostic agents against NTS_1_R-expressing tumors in the past few decades has been prompted both by the high incidence of NTS_1_R-positive cancer and by the high mortality rate of certain types thereof, e.g., PDAC [54,55]. Most often, at the time of diagnosis, PDAC has already spread in the body, owing to its asymptomatic character up to advanced stages in combination with the lack of effective diagnostic tools in its early stages. Hence, there is an urgent medical need for new molecular tools for the accurate and timely diagnosis of PDAC, including new NTS_1_R-directed SPECT or PET imaging agents [56]. At the same time, new effective therapeutic modalities need to become available in the clinic as soon as possible in an effort to increase the 5-year survival rate of PDAC patients well above the current 12–13% [54,55,56]. In this respect, NTS_1_R-specific carriers of beta, Auger e^−^ or alpha particle-emitting radiometals to cancer lesions are expected to significantly upgrade the existing anti-PDAC therapeutic arsenal with new powerful molecular tools. The rapid degradation of NT and NT(8–13)-based diagnostic/therapeutic agents in the biological milieu has been revealed as a major problem impacting efficacy. Therefore, we will next deliberate on the role of key enzymes involved in their fast in vivo break-down.

## 3. Metabolic Stability Considerations

The propensity of NT/NT(8–13)-based radioligands towards fast proteolytic degradation in the biological milieu became apparent in the early development stages due to its negative impact on tumor uptake [52]. Like most peptide radioligands, those based on NT(8–13) carry a radiometal chelate at the N-terminus too (Figure 1).

This N-capping modification inadvertently leads to aminopeptidase resistance [57,58]. Nevertheless, three major cleavage sites have been documented within the NT/NT(8–13) peptide chain: (i) Arg^8^–Arg^9^, (ii) Pro^10^–Tyr^11^ and (iii) Tyr^11^–Ile^12^. From this the combined role of a number of proteases has been assumed, as depicted in Figure 2 [49,59,60,61,62]. Firstly, the hydrolytic cleavage of the Arg^8^–Arg^9^ bond was attributed to thimet oligopeptidase (EC 3.4.24.15, EP24.15, THOP1), a neuropeptide-inactivating enzyme found in rat brain homogenates, including NT as a substrate [60,63,64]. THOP1 belongs to the M3 family of zinc-dependent endopeptidases, predominantly localized within the cell. So, the degradation of extracellular neuropeptides and hormones, such as NT, does not seem to be its main biological role [64,65,66]. Accordingly, it is reasonable to assume that THOP1 does not play a significant role in the degradation of NT(8–13) radioligands while in circulation and until they reach tumor sites. This setting is clearly distinct from metabolic events occurring in brain homogenates, where compartmentalization has been disrupted and THOP1 comes in direct contact with its substrates [65,67].

Secondly, neurolysin (EC 3.4.24.16, EP24.16, Nln) was shown to cleave the Pro^10^–Tyr^11^ bond. Nln is a zinc-dependent endopeptidase localized both within the cells and on the plasma membrane of cells in the brain, the liver and other peripheral tissues [68,69,70]. Unlike THOP1, Nln may come in direct contact with circulating NT(8–13) radioligands, and so its impact warrants further investigation with the aid of specific inhibitors [67,70].

Next, angiotensin converting enzyme (EC 3.4.15.1, peptidyl-dipeptidase A or 1, ACE) was shown to cleave the third Tyr^11^–Ile^12^ bond [62,71,72,73]. As a zinc metalloprotease, ACE cleaves a dipeptide from the C-terminus of its substrates, including NT and angiotensin I, and plays a role in blood pressure regulation through the renin–angiotensin system. ACE can also act as an endopeptidase, by the cleaving of peptides with amidated C-termini. ACE is found membrane-bound in many tissues, mainly in the lung and the kidneys, but is also present in the blood solute. Based on the above, the resistance of NT(8–13)-derived radioligands to ACE seems to play a very significant role in ensuring the adequate supply of intact radioligand molecules to tumor sites. The presence of ACE in blood allows the reliable assessment of NT(8–13)-based radioligand stability by the analysis of in vitro incubates of serum or plasma at 37 °C [52,74,75].

Lastly, a fourth protease has been shown to also participate in the cleavage of the last two Pro^10^–Tyr^11^ and Tyr^11^–Ile^12^ bonds of the NT/NT(8–13) chain, known as neutral endopeptidase (NEP, neprilysin, enkephalinase, CD10, CALLA, EC 3.4.24.11). NEP is a zinc metallopeptidase with a thermolysin-like specificity for cleaving peptides on the N-terminal side of hydrophobic residues. It is broadly expressed in often high local concentrations in the blood vasculature, the brain, the intestines and the kidneys, displaying a wide substrate repertoire, including NT and its analogs [50,52,59,60,76,77,78]. The fast hydrolytic action of NEP has evaded attention during typical in vitro stability studies in plasma or serum, where it is scarcely present because, as an ectoenzyme, it remains anchored on the membrane of epithelial cells. Interestingly, a number of recent reports have revealed its central role in compromising the stability and eventually the targeting efficacy of a multitude of peptide radioligands [67,79,80,81], including those based on NT(8–13) [82,83].

In view of the above, it is not surprising that the search for metabolically robust NT(8–13)-based radioligands has been quite intense in recent years, with the aim being to improve tumor targeting. Most interventions have been directed toward the aforementioned biodegradable bonds, or toward adjacent positions in the peptide chain. However, these interventions have often led to the impairment of other biological features of end radioligands, such as the loss of receptor affinity or unfavorable pharmacokinetics, as will be outlined in the following sections.

## 4. NT(7/8–13) Analogs Labeled with Tc-99m

### 4.1. The Tc(I)-Tricarbonyl Synthon Approach

Two major types of chelating systems were applied for labeling a number of NT(8–13) analogs with the preeminent SPECT radiometal Tc-99m [52,53]. A major part of this effort has been focused on the Tc(I)-tricarbonyl synthon methodology using N-terminally attached His (acting as a bidentate co-ligand) or *N*^α^-Ac-His (acting as a tridentate co-ligand) to complete the hexa-coordination sphere around the radiometal, as shown in Figure 3 [84]. A higher stability of the metal chelate was associated with the *N*^α^-Ac-His-coupled analogs, and therefore, this was the modification of choice in the design of second- and third-generation radioligands [84,85,86,87,88,89,90,91,92].

Several changes around the three identified biodegradable Arg^8^–Arg^9^, Pro^10^–Tyr^11^ and Tyr^11^–Ile^12^ bonds in NT(8–13) were pursued separately or in combination in such N-terminally His or *N*^α^-Ac-His coupled analogs (Table 1), and results related to NTS_1_R-affinity and tumor targeting in mice were investigated [52,53]. Efforts to stabilize the Arg^8^–Arg^9^ bond involved the replacement of either Arg by Lys in combination with the *N^α^*-methylation of one Arg residue or the reduction of the bond. The above changes were often combined with a Ile^12^→Tle^12^-replacement to additionally stabilize the Tyr^11^–Ile^12^ bond as well. The NT-I (His-NT(8–13) [85]) to NT-XIX (*N^α^*-His-Arg-NMeArg-Pro-Dmt-Tle-Leu, Dmt: 2,6-dimethyl-tyrosine [90,91]) series of analogs thus created yielded radioligands of clearly improved stabilities in serum incubates, with a metabolic stability half-life (t_1/2_) spanning from 10 min to 20 d [53].

Nevertheless, the so-acquired increase in radioligand in vitro stability might not reliably represent the actual in vivo stability, because the action of NEP is not taken into account [82,83]. For example, the derivatization of NT-I to NT-VI (*N^α^*-Ac-His-Lys(ψCH_2_NH)-Arg-Pro-Tyr-Ile-Leu) led to an increase in stability in serum t_1/2_ from 10–20 min to 3 h for [^99m^Tc]Tc-NT-VI, whereas the t_1/2_ determined in vivo did not surpass 2 min [87,92]. On the other hand, increases in tumor uptake and retention were often linked to abrupt increases of uptake and retention in the kidneys [53,85,87,88,89]. For example, [^99m^Tc]Tc-NT-VI [85], despite its better tumor uptake in mice, displayed an unfavorably high retention in the kidneys (4.7 ± 0.1% IA/g at 24 h pi) compared to its Arg^8^-counterpart, [^99m^Tc]Tc-NT-II (0.9 ± 0.7% IA/g at 24 h pi).

Eventually, NT-XI (*N^α^*-Ac-His-Lys(ψCH_2_NH)-Arg-Pro-Tyr-Tle-Leu) was the candidate of choice for clinical translation in a pilot study conducted in four PDAC patients [87,93]. Abdominal SPECT imaging with [^99m^Tc]Tc-NT-XI showed high uptake in the pathological lesion at the head of the pancreas in one patient at 3 h pi (Figure 4a), but failed to visualize confirmed lesions in the other 3 patients. Imaging findings were subsequently correlated with NTS_1_R expression levels determined by the autoradiography of surgically excised specimens from these lesions (Figure 4b). A high NTS_1_R expression was confirmed in the sample from the visualized tumor. A low cellularity was found in one patient with negative results on SPECT, whereas a total lack of NTS_1_R-expression was shown in the remaining two. This important study has highlighted the clinical value of NTS_1_R-directed radiodiagnostic tools in PDAC, and possibly other malignancies, to identify patients eligible for targeted radiotherapy with the respective radiotherapeutic analog.

In this respect, the two therapeutic rhenium radionuclides, Re-186 (half-life, t_1/2_ 3.7 d, E_β_-_max_ 1.07 and 0.94 MeV) and Re-188 (t_1/2_ 17 h, E_β_-_max_ 2.12 MeV), come into focus in view of the similarities of Tc and Re chemistries [94,95,96,97,98]. It should be noted that high purity and high specific activity [^188^Re]ReO_4_^−^, suitable for molecular targeting applications, is cost-effectively accessible via commercial [^188^W]W/[^188^Re]Re-generators [99]. The reduction of Re^+7^ in the generator eluate to a lower oxidation state (to +5, +3, or +1) allows coordination of the (radio)metal to suitable chelator systems, following the paradigm of Tc. This route provides the opportunity to introduce innovative [^99m^Tc]Tc/[^188^Re]Re-theranostic radiopharmaceutical pairs in clinical oncology [98]. However, practical challenges in the development of such theranostic pairs may arise from the notorious reluctance of rhenium to be reduced compared to technetium, and need to be competently addressed [100].

The first NT analog successfully labeled with Re-188 is NT-XI (Table 1). The resultant [^188^Re]Re-NT-XI was thoroughly studied in mice models in comparison with its [^99m^Tc]Tc-NT-XI counterpart [92]. This preliminary work further continued with the theranostic pair [^99m^Tc]Tc/[^188^Re]Re-NT-XIX (NT-XIX, *N^α^*-Ac-His-Arg-NMeArg-Pro-Dmt-Tle-Leu) [90,91]. Both radioligands displayed better metabolic stability not only in in vitro human serum incubates, but also in vivo (in vivo stability in mice blood t_1/2_ >1.2 h). They both displayed similar distribution patterns in mice bearing human colon adenocarcinoma HT-29 xenografts with favorable tumor-to-kidney ratios. This finding provided the impetus for a pilot therapy study in the same mice model with [^188^Re]Re-NT-XIX injected in either three or four fractionated doses. As a result, a 50% reduction in tumor growth could be achieved [91].

### 4.2. The Open-Chain Tetraamine Approach

#### 4.2.1. General

In another approach, an open-chain tetraamine chelator (6-carboxy-1,4,8,11-tetraazaundecane) was attached via a linker at the N-terminus of the NT(8–13) fragment and its analogs, yielding a series of NTS_1_R-directed [^99m^Tc]Tc-radioligands, the so-called “Demotensin” (DT) series [74,75]. Acyclic tetraamines have been proposed as a versatile means for labeling peptide analogs with Tc-99m, because they combine a number of attractive qualities for the effective targeting of high-affinity/low-capacity systems in the body [101]. They are known to form the monocationic octahedral *trans*-[Tc(V)(O)_2_(N_4_)]^+^ complexes with the four nitrogen donors at the equatorial plane and the two oxygens of the dioxo-Tc(V) core positioned at the apices (Figure 5) [102,103,104]. As a result, high hydrophilicity and kinetical inertness characterize this (radio)metal chelate, qualities reproduced at the tracer level, whereby Tc-99m can be bound to the tetraamine with high molar activities [101,103].

The initially introduced [^99m^Tc]Tc-Demotensin radioligands ([^99m^Tc]Tc-DT1 to [^99m^Tc]Tc-DT6; Table 2) displayed sub-nanomolar affinity to the human NTS_1_R, but their stability was shown to increase by key structural interventions operated on the NT(8–13) backbone, eventually resulting in better uptake in human colon adenocarcinoma HT-29 and/or WiDr xenografts in mice [74,75]. Specifically, in [^99m^Tc]Tc-DT1–[^99m^Tc]Tc-DT4, the tetraamine chelator was coupled to the peptide N-terminus via a Gly linker. Furthermore, the Arg^8^–Arg^9^ dipeptide in [^99m^Tc]Tc-DT1 was replaced by the reduced bond Lys^8^Ψ[CH_2_NH]Arg^9^ ([^99m^Tc]Tc-DT2 and [^99m^Tc]Tc-DT3) or Arg^8^Ψ[CH_2_NH]Arg^9^ ([^99m^Tc]Tc-DT4) constructs. In addition, an Ile^12^ to Tle^12^ switch was performed in [^99m^Tc]Tc-DT3 and [^99m^Tc]Tc-DT4 [75].

The stability in murine serum incubates was visibly improved in the Ile^12^→Tle^12^-substituted analogs, amongst which [^99m^Tc]Tc-DT4 also showed the highest stability in mouse urine collected 30 min pi. It is interesting to observe that [^99m^Tc]Tc-DT4 not only showed the best uptake in HT-29/WiDr xenografts (e.g., 4.72 ± 0.72%IA/g vs. 3.95 ± 0.64%IA/g of [^99m^Tc]Tc-DT3 in WiDr tumors at 1 h pi), but also a much lower kidney uptake and retention (16.29 ± 3.22%ΙA/g vs. 24.14 ± 4.16%IA/g of [^99m^Tc]Tc-DT3 at 1 h pi) [75]. Unlike the Lys^9^ analog ([^99m^Tc]Tc-DT3), [^99m^Tc]Tc-DT4 lacks a pendant primary amine, suspected to be related to the elevated kidney values.

A slightly different stabilization strategy was adopted in the case of [^99m^Tc]Tc-DT5 and [^99m^Tc]Tc-DT6, whereby the tetraamine unit was coupled to the N-peptide terminus via a *β-*Ala linker and Arg^9^ was replaced by Dab^9^ (diaminobutyric acid). Interestingly, this modification was previously reported for [Dab^9^]NT(8–13), one of the most potent NTS_1_R-agonists [105]. Furthermore, [^99m^Tc]Tc-DT6 underwent the additional Ile^12^→Tle^12^-substitution [74]. The preclinical evaluation of these two analogs revealed the improved serum stability of the Tle^12^-radioligand (94 ± 3% intact vs. 55.5 ± 5% of intact [^99m^Tc]Tc-DT5 at 15 min serum incubation), which translated into a significantly higher uptake in the WiDr implants (4.30 ± 0.45%IA/g of [^99m^Tc]Tc-DT6 vs. 1.68 ± 0.15%IA/g of [^99m^Tc]Tc-DT5 at 1 h pi). It is interesting to observe the unfavorably elevated kidney uptake of the more stable [^99m^Tc]Tc-DT6 (19.66 ± 2.92%IA/g vs. 13.51 ± 1.57%IA/g of [^99m^Tc]Tc-DT5 at 1 h pi) [74].

In order to explore the clinical prospects of the [^99m^Tc]Tc-DT series for NTS_1_R-targeted diagnostic tumor imaging, [^99m^Tc]Tc-DT6 was selected for a Pilot/Phase I study in 14 patients in advanced stages of PDAC, small- (SCLC) and non-small-cell lung cancer (NSCLC) or colon adenocarcinoma [106]. Unexpectedly, [^99m^Tc]Tc-DT6 visualized only brain metastases in three NSCLC patients, without visible localization in any of the other confirmed lesions. It should be noted, however, that none of the lesions were investigated for NTS_1_R-expression levels. Interestingly, although the stability of [^99m^Tc]Tc-DT6 in human serum incubates confirmed the preclinical stability results in mice, only a small portion of the radioligand was detected intact in the urine. Thus, the degradation of [^99m^Tc]Tc-DT6 in blood circulation could not be ruled out.

#### 4.2.2. In-Situ Stabilization Approaches

In a following study, the issue of in vivo stability was more carefully investigated with the aid of specific inhibitors against ACE and/or NEP, administered to mice [82]. Thus, the role of the above two proteases in the breakdown of [^99m^Tc]Tc-DT1, [^99m^Tc]Tc-DT5 and [^99m^Tc]Tc-DT6 in peripheral mice blood could be clearly demonstrated. Significant stability enhancements were registered following radioligand coinjection with the NEP-inhibitor phosphoramidon (PA) [107], the ACE-inhibitor lisinopril (Lis) [108], or a combination of the two [82]. Moreover, the impact of mice treatment with PA or/and Lis on the uptake in NTS_1_R-expressing xenografts could be clearly established.

For example, while only 1.8 ± 0.8% [^99m^Tc]Tc-DT1 remained intact in mice blood at 5 min pi, the coinjection of [^99m^Tc]Tc-DT1 with PA caused an increase to 26.4 ± 2.2% intact radioligand, and coinjection with Lis caused an increase to 18.8 ± 2.5%, while coinjection with both PA and Lis synergistically led to a stability increase to 72.3 ± 3.2% intact radioligand at 5 min pi. Notably, this in situ [^99m^Tc]Tc-DT1 stabilization translated into a significant enhancement of the respective uptake in WiDr xenografts at 4 h pi (from 1.20 ± 0.21%IA/g in controls to 4.58 ± 0.47%IA/g in PA-treated and to 9.60 ± 3.62%IA/g in PA+Lis-treated) [82]. At the same time, kidney uptake remained practically unaffected, resulting in higher tumor-to-kidney ratios and overall attractive pharmacokinetics, especially in the case of non-modified [^99m^Tc]Tc-DT1 (Figure 6).

A few interesting findings could be additionally derived from this study: (i) The Ile^12^→Tle^12^-replacement in [^99m^Tc]Tc-DT6 rendered the tracer practically resistant to ACE and thereby more stable in vivo (55.1 ± 3.9% intact at 5 min pi; controls). As a result, PA-treatment alone led to its almost full stabilization in peripheral mice blood (89.3 ± 6.7% intact at 5 min pi; PA-treated). (ii) On the other hand, the Ile^12^→Tle^12^-modification caused a substantial reduction in the in vitro uptake of [^99m^Tc]Tc-DT6 in WiDr cells, compared with the other two Ile^12^ radiotracers ([^99m^Tc]Tc-DT1 and [^99m^Tc]Tc-DT5). (iii) [^99m^Tc]Tc-DT6 ended up with the lowest uptake in the WiDr xenografts, despite its higher in vivo stability, presumably as a result of its compromised uptake in the cells. (iv) The Dab^9^-modified [^99m^Tc]Tc-DT5 and [^99m^Tc]Tc-DT6 displayed significantly higher kidney values compared to the [^99m^Tc]Tc-DT1 prototype, lacking the pendant primary amine of Dab^9^ [82].

Quite comparable results were obtained when [^99m^Tc]Tc-DT1 and [^99m^Tc]Tc-DT5 were studied in pancreatic AsPC-1 cells in vitro [83]. In the stability studies, however, in situ NEP inhibition was achieved with the aid of the potent and specific NEP-inhibitor sacubitrilat (LBQ657) instead of PA. The registered anti-hypertensive drug Entresto^®^, containing the sacubitril (AHU377) precursor, was used as the source of sacubitrilat [109,110,111,112]. In the protocol, mice received a predetermined dose of Entresto^®^ by oral gavage 30 min prior to radioligand intravenous (iv) injection. This period of time allowed for the absorption and hydrolysis of sacubitril by plasma proteases to sacubitrilat, which actually effected the in-situ inhibition of NEP [83,111,112,113]. Notably, stabilization effects were very comparable with those of PA (Figure 7—for comparison with Figure 6) [82,83]. Specifically, the radiotracer remained 63.8 ± 7.5% intact in the circulation of mice treated with Entresto^®^+Lis at 5 min vs. untreated mice with the in-situ stabilization of [^99m^Tc]Tc-DT1 (1.8 ± 0.8% intact), leading to significant increases in radioligand uptake in the AsPC-1 xenografts at 4 h pi (1.25 ± 0.14%IA/g in untreated controls to 7.05 ± 0.80%IA/g in Entresto^®^+Lis-treated mice). The successful application of registered drugs in the stabilization of biodegradable NT radioligands with this protocol turned out to be a promising new approach, which warrants further validation at the clinical level.

Understandably, the clinical translation of a radiotracer administered in combination with two additional drugs is expected to overcome regulatory and practical challenges related to the approval of clinical protocols, even when strictly registered and widely used drugs are considered for application. In fact, dual-peptidase inhibitors proposed as anti-hypertensive drugs for long-term use, such as the dual NEP/ACE-inhibitor omapatrilat, have been linked to higher side-effects [114,115]. It should be noted that omapatrilat was eventually discontinued due to increased biosafety problems, especially as regards angioedema [116]. In view of these concerns, further chemical modifications on the [^99m^Tc]Tc-DT1 prototype were subsequently pursued. The objective was instead set to minimize or altogether circumvent the need for combined or single protease inhibitor regimens, even when meant for a single application only and not for chronic use.

#### 4.2.3. C-Terminus Modifications

To begin with, the method of C-terminal DAsn-elongation [117] was pursued, yielding [^99m^Tc]Tc-DT7 (DT7, [DAsn^14^]DT1); DT7 showed decreased NTS_1_R-affinity (IC_50_ = 29.1 *±* 4.1 nM) [117]. In addition, the C-terminal “homologation” route was adopted [118,119,120,121]. Specifically, Leu^13^ was replaced with *β*^3^-homoleucine to develop [^99m^Tc]Tc-DT8 (DT8, [*β*^3^-hLeu^13^]DT1) [118]. Unlabeled DT8 showed sub-nanomolar affinity to the human NTS_1_R (IC_50_ = 0.6 *±* 0.1 nM). However, neither [^99m^Tc]Tc-DT7 nor [^99m^Tc]Tc-DT8 could internalize or be taken up by AsPC-1 cells. In peripheral mice blood, they were less degraded than [^99m^Tc]Tc-DT1 (20–28% intact vs. <2% intact, respectively, at 5 min pi). The pretreatment of mice with Entresto^®^ led to the significant enhancement of metabolic stability for both radioligands (60–65% intact at 5 min pi), which did not significantly increase in the Entresto^®^+Lis-treated mice [118]. This result implies that C-terminal DAsn-elongation and *β*-homoleucine “homologation” were successful in rendering [^99m^Tc]Tc-DT7/8 resistant to ACE, but not to NEP [118]. A similar finding was reported for a Ga-68 labeled DOTA (1,4,7,10-tetraazacyclododecane-1,4,7,10-tetraacetic acid) coupled analog, likewise derivatized at position 13 to *β*-homoleucine^13^, which showed high in vitro stability, but rapidly degraded in mouse circulation (vide infra) [121].

#### 4.2.4. Introduction of Lateral Chains

Ultimately, a new effort to increase metabolic stability, while preserving receptor affinity, cell uptake, tumor targeting and favorable pharmacokinetics, involved the substitution of Pro^7^ in the [^99m^Tc]Tc-DT1 motif by Lys^7^ and the attachment of a lateral chain at its *ε*-NH_2_ [118,122]. This route was inspired by the 16-mer invertebrate counterpart of mammalian NT, contulakin-G (pGlu-Ser-Glu-Glu-Gly-Gly-Ser-Asn-Ala-Thr(R)-Lys-Lys-Pro-Tyr-Ile-Leu-OH), which carries a disaccharide (R, beta-D-Galp-(1→3)-alpha-D-GalpNAc-(1→); Galp, galactopyranosyl and GalpNAc, N-acetylgalactosaminepyranosyl) at Thr^10^, a position confirmed to be equivalent to Pro^7^ in NT (Figure 8) [123].

Notably, a number of different side chains were attached in either contulakin-G (Thr^10^) or NT (Pro^7^), leading to analogs of high receptor-affinity and enhanced bioavailability [123,124,125,126]. Pendant groups included palmitoyl as well as other albumin-binding entities (ABE) or non-albumin-binding chains, like polyethyleneglycol (PEG_X_) groups [127,128,129,130].

In line with the above, the first branched [^99m^Tc]Tc-DT1 mimic turned out to be [^99m^Tc]Tc-DT9 (Figure 9, Table 2), carrying a palmitoyl chain at Lys^7^ [118].

Indeed, [^99m^Tc]Tc-DT9 displayed a high receptor affinity and internalization rate in AsPC-1 cells combined with exceptionally high in vivo stability. However, the pharmacokinetics in mice turned out to be unfavorable, with high background activity levels in most healthy tissues and excretory organs. This result was tentatively assigned to the high albumin binding of the lipid acid chain in combination with the marked lipophilicity boost it conveyed [118]. These positive findings, with the exception of the unfavorable increase in background radioactivity levels of [^99m^Tc]Tc-DT9, have nevertheless confirmed the suitability of position seven to hosting long pendant chains [123,124,125,126]. Based on these conclusions, a second generation of branched [^99m^Tc]Tc-DT1 mimics was developed, carrying less lipophilic, ABE or/and PEGx lateral chains instead of palmitoyl, aiming to improve pharmacokinetics (Figure 9; Table 2) [122].

**Table 2:** NT analogs of the NT(8–13) fragment conjugated to the N_4_ chelator via a linker, enabling labeling with Tc-99m. The compounds in the table are reported as the Demotensin (DT) series of analogs.

Code Name	Chelator	Linker	Sequence	C-Terminus	References
NT(8–13)	-	-	H-Arg-Arg-Pro-Tyr-Ile-Leu-OH	-	
DT1	N_4_-	-Gly-	-Arg-Arg-Pro-Tyr-Ile-Leu-OH	-	[74,75,82]
DT2	N_4_-	-Gly-	-Lys-Ψ(CH_2_NH)-Arg-Pro-Tyr-Ile-Leu-OH	-	[75,82,83]
DT3	N_4_-	-Gly-	-Lys-Ψ(CH_2_NH)-Arg-Pro-Tyr-Tle-Leu-OH	-	[75,82,83]
DT4	N_4_-	-Gly-	-Arg-Ψ(CH_2_NH)-Arg-Pro-Tyr-Tle-Leu-OH	-	[75,82,83]
DT5	N_4_-	-*β-*Ala-	-Arg-Dab-Pro-Tyr-Ile-Leu-OH	-	[74,82,83]
DT6	N_4_-	-*β-*Ala-	-Arg-Dab-Pro-Tyr-Tle-Leu-OH	-	[74,82,83,106]
DT7	N_4_-	-Gly-	-Arg-Arg-Pro-Tyr-Ile-Leu-	-DAsn-OH	[118]
DT8	N_4_-	-Gly-	-Arg-Arg-Pro-Tyr-Ile-Leu-	-*β*^3^-hLeu-OH	[118]
DT9	N_4_-	-Lys(palmitoyl)-	-Arg-Arg-Pro-Tyr-Ile-Leu-OH	-	[118]
DT10	N_4_-	-Lys(MPBA)-	-Arg-Arg-Pro-Tyr-Ile-Leu-OH	-	[118,122]
DT11	N_4_-	-Lys(PEG4-MPBA)-	-Arg-Arg-Pro-Tyr-Ile-Leu-OH	-	[122]
DT12	N_4_-	-Lys(CO-CH_2_-PEG5-OMe)-	-Arg-Arg-Pro-Tyr-Ile-Leu-OH	-	[122]

Tle, *tert*-leucine; *β-*Ala, beta-alanine; Dab, 2,4-Diaminobutyric acid; *β*^3^-hLeu-OH, *β*^3^-homoleucine or 3-amino-5-methylhexanoic acid; MPBA, 4-(4-methylphenyl)butyric acid; PEG4, Amino-PEG4-acid, PEG, polyethyleneglycol. The Linkers in DT9-DT12 are separately depicted in Figure 9.

Steric factors (lateral chain length) and binding to albumin (in the case of ABE) were found to contribute to stability increases and to impact the internalization rates, tumor uptake and pharmacokinetics of resultant analogs. The most attractive features were displayed by [^99m^Tc]Tc-DT11, whereby an ABE (MPBA, 4-(4-methylphenyl)butyric acid) was coupled to the *ε*-NH_2_ of Lys^7^ via a PEG4 linker (14-amino-3,6,9,12-tetraoxatetradecan-1-oic acid) [122]. The improved in vivo stability of [^99m^Tc]Tc-DT11 (56.6 ± 5.2% intact in controls vs. 77.0 ± 3.31% intact in Entresto^®^-treated samples at 5 min pi) was combined with fast cell internalization, leading to the highest uptake in AsPC-1 tumors in mice, not only during single NEP inhibition regimens (6.14 ± 0.08%IA/g at 4 h pi), but also when injected alone (4.48 ± 0.37%IA/g at 4 h pi), qualifying for further validation in patients with NTS_1_R-positive tumors with SPECT/CT.

In conclusion, the search for a clinically effective Tc-99m radiotracer in the diagnostic imaging of NTS_1_R-expressing human tumors has been quite intense, and has very recently yielded promising results. Besides the wide and cost-effective availability of Tc-99m, in excellent quality, in hospitals [101], the recent technological advances in SPECT and SPECT/CT instrumentation [131,132,133,134], have rendered this effort worth taking. On the other hand, the concept of radiotheranostics with the aid of [^99m^Tc]Tc/[^186/188^Re]Re-NT-based pairs is still in its infancy today. Nevertheless, the similarities of Tc and Re chemistries on one hand [94,95,96,97] and the availability of commercial [^188^W]W/[^188^Re]Re-generators on the other [98,99] offer new exciting prospects in this regard, with the first efforts toward this goal having already been made [90,91,92].

## 5. Bi/Trivalent Radiometal-Tagged NT Analogs

### 5.1. General

A wide panel of bi/trivalent radiometals have been increasing in availability for use in theranostic applications in recent years [11,12,13,14,15,16,17,135]. These include positron emitters for PET imaging (Ga-68, Cu-64, Zr-89, Y-86, Co-55), gamma emitters for SPECT (In-111) and particle emitters for radiotherapy (Lu-177, Y-90, Cu-67, Tb-161, Bi-213, Ac-225, Pb-212). Thus, acyclic, macrocyclic and hybrid polyamine–polycarboxylates have served as chelators to label a variety of vectors with these theranostic radiometals (Figure 10).

The selection of the appropriate radiometal depends on several factors, like its decay characteristics, its availability and coordination chemistry, and the intended application, along with critical physicochemical properties of the vector [11,12,13,14,136,137,138].

**Figure 10:**
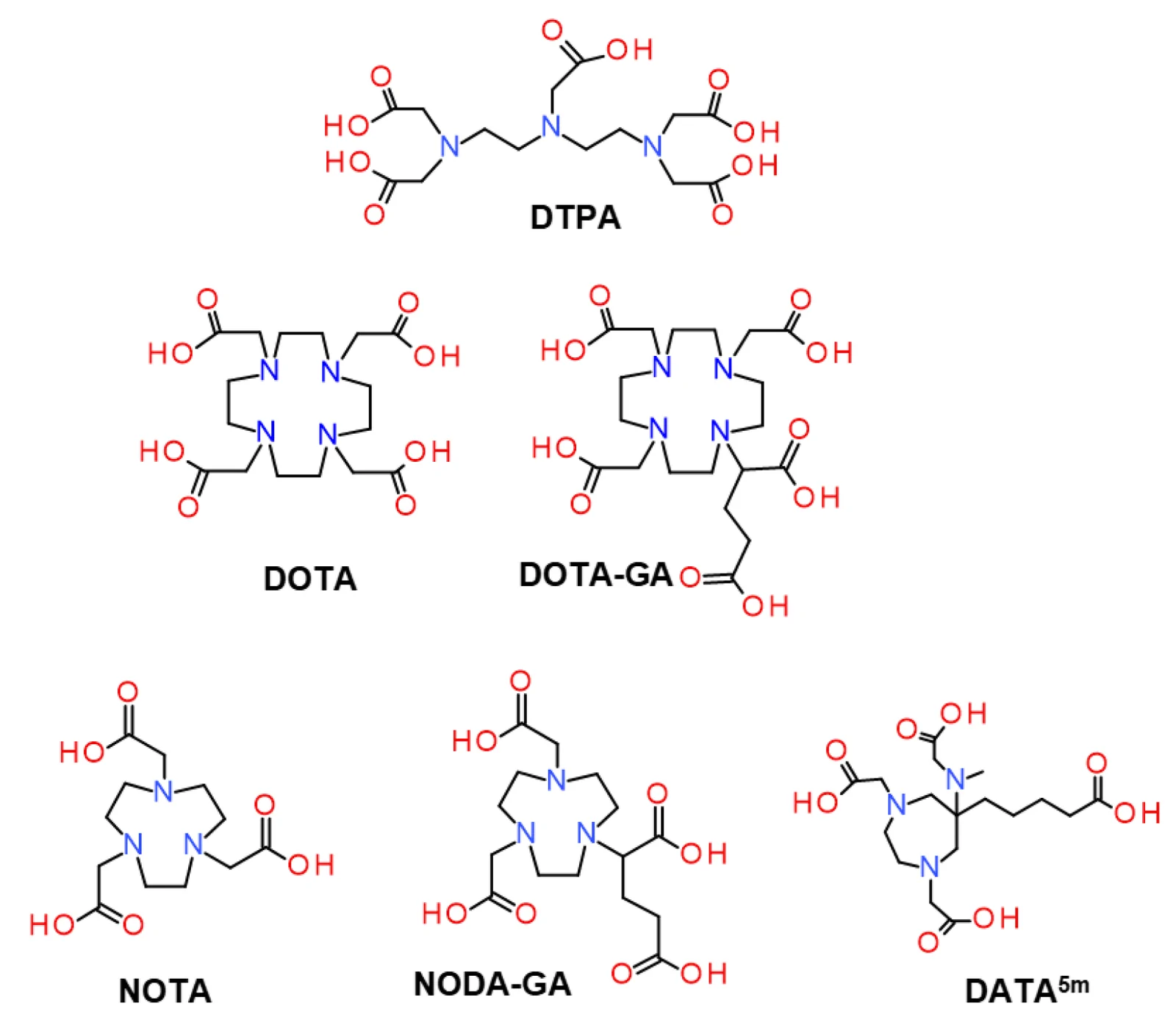
Examples of known bifunctional chelators for bi- and trivalent (radio)metals. DTPA, diethylenetriaminepentaacetic acid; DOTA, 1,4,7,10-tetraazacyclododecane-1,4,7,10-tetraacetic acid; DOTA-GA, 1,4,7,10-tetraazacyclododecane-1-(glutaric acid)-4,7,10-triacetic acid; NOTA, 1,4,7-triazacyclononane-1,4,7-triacetic acid; NODA-GA, 1,4,7-triazacyclononane-1-glutaric acid-4,7-acetic acid; DATA^5m^, (6-pentanoic acid)-6-(amino)methy-1,4-diazepinetriacetate.

### 5.2. First DTPA/DOTA-Coupled NT(6–13) Radioligands

A first systematic structure–activity relationship study was conducted with a series of NT(6–13) analogs, with the Arg^8^, Arg^9^, or/and Ile^12^ residues substituted by several commercially available amino acid mimics. Originally, DTPA (diethylenetriaminepentaacetic acid) was attached at their N-terminus for labeling with In-111 (Table 3). The analogs were screened for their NTS_1_R affinity in HT-29 cell membrane homogenates and for their 4 h stability in 37 °C human serum incubates, and also in urine collected from rats [139]. As a result, [^111^In]In-MP2530 ([^111^In]In-DTPA-Gly(Pip)-Pro-Gly(PipAm)-Arg-Pro-Tyr-*t*BuGly-Leu-OH; Gly(Pip), 4-piperidinyl-*S*-glycine; Gly(PipAm), 4-piperidinyl-(*N*-amidino)-*S*-glycine; *t*BuGly, *tert-*butyl-*S*-glycine identical to Tle) was the radioligand of choice for further in vivo evaluation in mice bearing HT-29 xenografts. [^111^In]In-MP2530 achieved fair NTS_1_R-mediated uptake and good retention in the mice-grafted tumors (2.8 ± 0.7%IA/g at 1 h pi, 2.2 ± 0.1%IA/g at 4 h pi) against a clear background, with the exception of the kidneys. Uptake in the kidneys turned out to be 2–3-fold higher than in the tumors.

It should be noted that the metabolic stability of [^111^In]In-MP2530 was determined in vitro, with no in vivo data offered. Hence, the involvement of NEP and possibly other proteases in a potentially fast breakdown of circulating [^111^In]In-MP2530 was overlooked. Radioligand in vivo degradation by NEP is a crucial event, because it is expected to compromise the supply of intact [^111^In]In-MP2530 to the implanted tumor [82,83,139]. Nevertheless, [^111^In]In-MP2530 was next studied in a number of animal models in an effort to better understand the mechanisms underlying its uptake and retention in tumors and kidneys [140,141,142,143]. Eventually, the metabolic stability of [^111^In]In-MP2530 as well as its DOTA-coupled version [^111^In]In-MP2656 ([^111^In]In-DOTA-Gly(Pip)-Pro-Gly(PipAm)-Arg-Pro-Tyr-*t*BuGly-Leu-OH) were investigated in peripheral mice blood at 30 min pi. They were found to be much lower than their in vitro stability, in support of the NEP involvement hypothesis [143].

Unfortunately, the limited efficacy of [^111^In]In-MP2530 in targeting NTS_1_R-positive tumors in mice was next manifested in patients too. A pilot study was conducted with [^111^In]In-MP2530 in 13 patients with suspected pancreatic adenocarcinoma, which was confirmed in 7. [^111^In]In-MP2530 failed to visualize the tumors in six out of these seven confirmed patients, which showed low NTS_1_R expression levels during receptor autoradiography. Most importantly, [^111^In]In-MP2530 failed to visualize even the NTS_1_R-rich lesion in the seventh patient. This disappointing outcome was attributed to the poor in vivo stability of [^111^In]In-MP2530, eventually compromising its delivery and pursuant uptake in the single NTS_1_R-rich lesion. This assumption was further supported by the HPLC analysis of patient plasma collected 10 min pi, showing a pattern dominated by [^111^In]In-MP2530-related radiometabolites [144]. Hence, the substitution of DTPA by DOTA in [^111^In]In-MP2530, to allow labeling with therapeutic radiometals (like Y-90 or Lu-177) for the pursuit of anti-pancreatic cancer radiotherapy, was abandoned. Priorities were instead set on the development of NTS_1_R-seeking peptide analogs conjugated to DOTA that remain metabolically robust in vivo [144].

The impact of chelator type and linker (X) length on the biological performance of DOTA-X-[*N^α^*-(N-Me)Arg^8^,Dmt^11^,Tle^12^]NT(6–13) analogs (DOTA-X-Lys-Pro-(N-Me)Arg-Arg-Pro-Dmt-Tle-Leu-OH) labeled with Lu-177 was investigated using HT-29 cells and mice models (Table 3) [145]. The NTS_1_R affinity of metal-free and [^nat^/^177^Lu]Lu-tagged conjugates was found dependent on X length. While greatly reduced in the absence of a linker (X = 0; IC_50_ ≈ 50 nM), the receptor affinity was restored in the four-atom hydrocarbon linker (X = *β-*Ala; IC_50_ < 30 nM) and beyond [145]. Thus, the [^177^Lu]Lu-DOTA-*β-*Ala-[*N^α^*-(N-Me)Arg^8^,Dmt^11^,Tle^12^]NT(6–13) analog showed high in vitro stability in human serum, but the in vivo stability was not determined. A good uptake was found in the HT-29 xenografts in mice (3.1 ± 0.4%IA/g at 4 h pi), but the kidney uptake was higher (17.0 ± 2.2%IA/g) [145].

The effect of the linker type and length was also investigated in a similar experimental setting using a series of DOTA-X-NT(8–13) analogs, likewise labeled with Lu-177 (Table 3) [146]. The study revealed that the [^177^Lu]Lu-radioligands with X = PEG4 or Ahx (aminohexanoic acid) showed improved receptor affinity and internalization in HT-29 cells compared with their counterpart without a linker (X = 0). In this case, however, all radioligands were rapidly degraded in human plasma incubates (t_1/2_ < 5 min) [146]. In an effort to improve metabolic stability, a series of DOTA-PEG4-NT(8–13) analogs underwent Arg^8^ or/and Arg^9^ replacement(s) with the respective Lys combined with Tle^12^ substitutions, labeled with Lu-177 and studied for receptor affinity, internalization and serum stability [147]. Unsurprisingly, the metabolic stability in serum was enhanced in all Tle^12^-substituted analogs, but at the cost of NTS_1_R affinity and internalization [147]. Interestingly, this finding is in line with similar observations on Tle^12^-analogs in the [^99m^Tc]Tc-DT series [82,83]. Although the study did not offer any animal data, it once again confirmed the superiority of [^177^Lu]Lu-DOTA-PEG4-NT(8–13) vs. the [^177^Lu]Lu-DOTA-NT(8–13) motif in terms of NTS_1_R affinity, cell internalization and serum stability [147].

### 5.3. Triazolyl-Substituted NT(8–13) Radioligands

In an alternative approach, the improvement of metabolic stability was pursued by replacing key amide bond(s) in [^177^Lu]Lu-DOTA-PEG4-NT(8–13) and its Tle^12^-substituted version with 1,2,3-triazole isostere(s) (Figure 11) [148,149].

A systematic “triazole scan” study revealed that both the Ile^12^ and Tle^12^ radioligands that underwent the Arg-Ψ[Tz]-Arg substitution are stable in human serum for at least 4 h of incubation at 37 °C, without reporting results from in vivo experiments [148]. Only the Ile^12^ version retained a single-digit affinity for the NTS_1_R, whereas the Tle^12^-substituted analog showed a substantial drop in receptor affinity (K_D_ > 200 nM) [148]. Biodistribution profiles were compared in mice bearing HT-29 xenografts, revealing uptake in the implants or the kidneys not surpassing 2.5%IA/g between 1 and 4 h pi for any of the tested radioligands.

Interestingly though, the compounds that underwent the PEG4-ψ[Tz]-Arg switch, despite their low receptor affinity and internalization in HT-29 cells, reached similar tumor uptake to their Arg-ψ[Tz]-Arg switch counterparts, presumably due to their significantly higher stability in human serum [148]. Based on this finding, the in vitro properties of double-substituted PEG4-ψ[Tz]-Arg-ψ[Tz]-Arg analogs were compared with those of their single PEG4-ψ[Tz]-Arg or Arg-ψ[Tz]-Arg analogs; compounds were divided into those unmodified at position 12 and those that were Tle^12^-substituted [148]. The effect of Ile^12^→Tle^12^-substitution was consistently found to have the most profound effect in improving serum stability, but at the cost of NTS_1_R-affinity and internalization in HT-29 cells. In contrast, the introduction of triazoles had a less negative impact on the latter qualities [149].

### 5.4. Mimics of the NT-I/XIX Series

By replacing the *N^a^*-Ac-His chelator (for Tc-99m or Re-188) in NT-VI and NT-XI (Table 1) [85,87,92] by DTPA, DTPA-NT-VI (DTPA-Lys-Ψ(CH_2_NH)-Arg-Pro-Tyr-Ile-Leu) and DTPA-NT-XI (DTPA-Lys-Ψ(CH_2_NH)-Arg-Pro-Tyr-Tle-Leu) were obtained, both of which are suitable for labeling with In-111 (Table 4) [150]. The in vivo stability of the new radioligands was tested at 15 min pi and turned out to be superior for [^111^In]In-DTPA-NT-XI (47% intact; Tle^12^-analog) compared to [^111^In]In-DTPA-NT-VI (10% intact; Ile^12^-analog) [150]. The uptake of either analog in HT-29 xenografts in mice was very low, even at 1 h pi ([^111^In]In-DTPA-NT-VI—0.62 ± 0.06%IA/g; [^111^In]In-DTPA-NT-XI—0.52 ± 0.23%IA/g). In addition, kidney uptake ([^111^In]In-DTPA-NT-VI—2.80 ± 0.37%IA/g; [^111^In]In-DTPA-NT-XI—3.90 ± 0.59%IA/g) was unfavorably higher than tumor uptake. Compared to their Tc-99m counterparts ([^99m^Tc]Tc-NT-VI—1.7 ± 0.3%IA/g [84]; ([^99m^Tc]Tc-NT-XI—3.9 ± 0.7%IA/g at 1.5 h pi [87]), the two [^111^In]In-DTPA-modified analogs showed inferior tumor uptake. This finding underscores the significant impact of the (radio)metal chelate moiety on the biological profile of small peptide vectors.

### 5.5. Peptoid-like Substitutions at the N-Terminus

Another interesting approach toward the improved metabolic stability of radioligands based on the Tle^12^-modified NT(8–13) fragment was the replacement of the N-terminal Arg^8^–Arg^9^ dipeptide by the peptoid-like construct N-(4-aminobutyl)Gly-Lys (Nlys-Lys) [119]. Initially, the DOTA chelator was coupled at the N-terminus and the resulting conjugate (NT’-1, DOTA-Nlys-Lys-Pro-Tyr-Tle-Leu-OH; Table 4) was labeled with the PET radionuclide Ga-68 [151]. A few interesting findings were acquired by the evaluation of this peptoid–peptide hybrid analog and its Ga-68-labeled version, [^68^Ga]Ga-NT’-1. Firstly, the attachment of DOTA to the N-terminus resulted in a profoundly reduced NTS_1_R affinity (2300 ± 1000 nM), but after coordination with Ga-68, the affinity drastically increased (180 ± 47 nM). This finding highlights the remarkable impact of the charge, type and position of the chelator and metal chelate on its interaction capabilities with the NTS_1_R-target. Next, the radiopeptide achieved a high in vitro stability in human serum, and remained >90% intact in mouse blood circulation at 10 min pi. [^68^Ga]Ga-NT’-1 nevertheless achieved low but partially NTS_1_R-mediated uptake in HT-29 xenografts in mice (0.72 ± 0.18% IA/g vs. 0.34 ± 0.17% IA/g in block at 65 min pi). At the same time, renal values were disadvantageously high at this time point (8.1% IA/g, >10-fold higher than tumor values), most probably due to the presence of two pendant primary amine groups in the molecule [151].

In view of the above, a series of -Nlys-Lys-Pro-Tyr-Tle-Leu-based analogs were subsequently developed, with the DOTA or the NODAGA chelator coupled at the N-terminus via different linkers (Table 4) [152]. The chelator-linker combination directly impacted the NTS_1_R-affinity of the metal-free analogs. Interestingly though, the binding of Ga led to a striking improvement in the affinity of all (radio)Ga-tagged analogs to the NTS_1_R. Eventually, [^68^Ga]Ga-NT’-5 (the DOTA-Lys- analog; Table 4) and [^68^Ga]Ga-NT’-7, (the NODAGA-PEG6-Lys- analog; Table 4) ended up with better and similar affinities for the NTS_1_R (19 ± 6.4 and 20 ± 1.2 nM, respectively) [152]. Thus, they were both selected for further evaluation in HT-29 xenograft-bearing mice. In this study, they displayed similar and NTS_1_R-mediated tumor uptakes at 1 h pi ([^68^Ga]Ga-NT’-5—1.40 ± 0.13%IA/g vs. 0.45 ± 0.19%IA/g in block; and [^68^Ga]Ga-NT’-7—1.55 ± 0.35%IA/g vs. 0.21 ± 0.06%IA/g in block), which were clearly improved compared with [^68^Ga]Ga-NT’-1, altogether lacking a linker [151,152]. Again, renal values were by far higher than tumor values ([^68^Ga]Ga-NT’-5: 34.87 ± 2.48%IA/g and [^68^Ga]Ga-NT’-7: 44.59 ± 6.46%IA/g), in line with the negative influence of the two pendant primary amines of the Nlys-Lys segment [152].

### 5.6. Terminal Homologation

An alternative way to increase the resistance of bioactive peptides and their analogs to the proteolytic action of exopeptidases is the “terminal homologation” route. In this method, the terminal *a*-amino acid residues in a peptide are replaced by *β*-amino acids. In the case of NT(8–13), this route yielded single C-*β*^3^-hLeu-subsituted or double N-*β^2^*-hArg-/C-*β^3^*-hLeu-substituted analogs with prolonged stability in human serum, while preserving single-digit nanomolar affinity to the NTS_1_R [117,119,120,121]. The coupling of DOTA to their N-terminus directly or via a series of linkers (Table 4) drastically reduced NTS_1_R affinity (82–410 nM), although molecular simulations predicted favorable fitting to the receptor [121]. The in vitro stability of the Ga-68 radiolabeled analogs in human serum was high for at least 1 h, implying a fair resistance to ACE [82,118]. In contrast, the analysis of mice blood samples collected 10–15 min pi revealed the presence of hydrophilic radiometabolic entities with only traces of the original radioligands. This finding is concordant with the action of NEP, and possibly other proteases, in their fast breakdown in circulation [82,118]. [^68^Ga]Ga-sc.sn.h-NT (sc.sn.h-NT, DOTA-*β*-hGly-Arg-Arg-Pro-Tyr-Ile-*β^3^*-hLeu) and [^68^Ga]Ga-sc.dn.h-NT (sc.dn.h-NT, DOTA-*β*-hGly-*β^2^*-h(*R*)Arg-Arg-Pro-Tyr-Ile-*β^3^*-hLeu) (Table 4) were studied next in mice bearing HT-29 xenografts. As expected by the sub-optimal receptor affinity and the fast in vivo degradation, the tumor uptake of these analogs was low at 1 h pi. Nevertheless, this uptake was shown to be NTS_1_R-mediated at least in part, especially in the case of [^68^Ga]Ga-sc.sn.h-NT (1.5 ± 1.0%IA/g vs. 0.6 ± 0.2%IA/g in block) [121]. It should be mentioned, that kidney values, although surpassing tumor values, remained <3%IA/g. Interestingly, neither analog carries pendant primary amine groups, in contrast to the previous series of Nlys-Lys-modified radioligands (Table 4) [121,151,152].

**Table 4:** Analogs of the NT(6/8–13) fragment conjugated to different chelators for bi/trivalent radiometals, either directly or via a linker; non-natural amino acids and linkers are depicted for convenience at the head of the table.

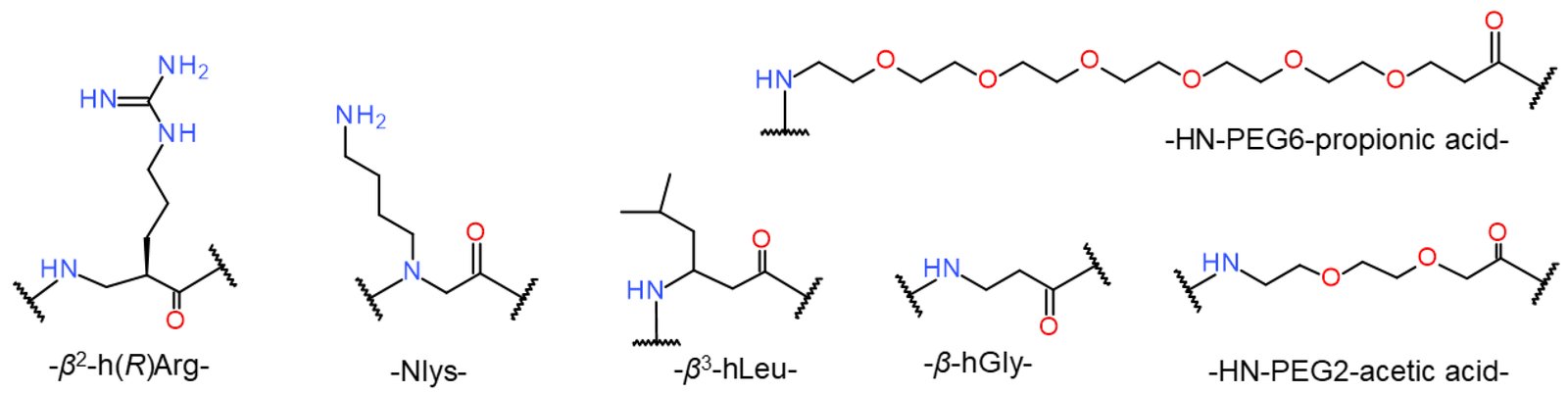				
Code Name	Chelator	Linker	Sequence	References
NT(6–13)	-	-	H-Lys-Pro-Arg-Arg-Pro-Tyr-Ile-Leu-OH	
DTPA-NT-VI	DTPA-	-	-Lys-Ψ(CH_2_NH)-Arg-Pro-Tyr-Ile-Leu-OH	[150]
DTPA-NT-XI	DTPA-	-	-Lys-Ψ(CH_2_NH)-Arg-Pro-Tyr-Tle-Leu-OH	[150]
NT’-1	DOTA-	-	-Nlys-Lys-Pro-Tyr-Tle-Leu-OH	[151,152]
NT’-3	NODAGA-	-PEG6*-	-Nlys-Lys-Pro-Tyr-Tle-Leu-OH	[152]
NT’-5	DOTA-	-Lys-	-Nlys-Lys-Pro-Tyr-Tle-Leu-OH	[152]
NT’-7	NODAGA-	-PEG6-Lys-	-Nlys-Lys-Pro-Tyr-Tle-Leu-OH	[152]
s.ch-NT	DOTA-	-*β*-hGly-	-Arg-Arg-Pro-Tyr-Ile-*β*^3^-hLeu-OH	[120,121]
sc.sn.h-NT	DOTA-	-*β*-hGly-	-Arg-Arg-Pro-Tyr-Ile-*β*^3^-hLeu-OH	[120,121]
sc.dn.h-NT	DOTA-	-	-*β*^2^-h(*R*)Arg-Arg-Pro-Tyr-Ile-*β*^3^-hLeu-OH	[120,121]

Nlys-Lys, N-(4-aminobutyl)Gly-Lys; Tle, *tert*-Leu; PEG6*, 21-amino-4,7,10,13,16,19-hexaoxaheneicosanoic acid, or amino-PEG6-propionic acid; s.ch, single C-terminal homologated; *β^3^*-hLeu, beta-3 homoleucine; sc.sn.h-, single C-terminal/single N-terminal homologated; *β*-hGly, beta-homoglycine; sc.dn.h-, single C-terminal/double N-terminal homologated; *β^2^*-h(*R*)Arg, beta-2 homoarginine.

### 5.7. Silylated Analogs

Another interesting route toward the metabolic stability enhancement of the NT(8–13) fragment has involved the single or double replacement of Ile^12^ or/and Leu^13^ in the H-Lys-Lys-Arg-Pro-Tyr-Ile-Leu-OH (JMV438) chain by the unnatural silicon-containing TMSAla-OH (L-(trimethylsilyl)alanine), giving rise to a small number of C-terminal silylated analogs [153,154]. Among those, JMV2007, carrying TMSAla-OH at position 13, was the only one displaying sub-nanomolar affinity for NTS_1_R (0.02 ± 0.002 nM vs. 0.82 ± 0.03 nM of the non-modified NT(8–13) fragment). Unlike JMV2007, the TMSAla-OH^12^-substituted JMV2006 showed a drastic loss of affinity to the NTS_1_R (93.8 ± 3.5 nM) [153,154]. Based on these findings, the chelator DOTA was then attached at the N-terminus of JMV2007, JMV2006 or the non-silylated analog JMV438, as well as of the NT(8–13) reference via the APAC (2-(4-aminopiperidin-1-yl)acetic acid) linker. Accordingly, a series of DOTA-conjugates was generated (Table 5). They were subsequently labeled with Ga-68 and their biological properties were compared in HT-29 cell and mice models [153,154]. The coupling of DOTA had a negative effect on affinity, although a basic linker was introduced between the chelator and the N-terminus; the coordination of Ga partially “repaired” NTS_1_R-affinity. The best performing analog turned out to be JMV6659, namely, the TMSAla-OH^13^-substituted one (34.5 ± 1.4 nM). In line with previous observations, the respective Ga-tagged version of JMV6659 showed better affinity, which was the highest in this series of analogs (6.29 ± 1.37 nM) [154]. Although the two silylated analogs, [^68^Ga]Ga-JMV6659 and [^68^Ga]Ga-JMV6658, displayed higher stability in serum incubates at 37 °C after 15 min, none survived in serum after 45 min. Sadly, results for in vivo stability were not provided [154]. Eventually, the most promising [^68^Ga]Ga-JMV6659 analog did achieve NTS_1_R-specific uptake in the HT-29 tumors at 2 h pi in mice (7.80 ± 0.54%IA/g vs. 1.38 ± 0.71%IA/g in block). In this case again, kidney values were unfavorably higher than tumor values (19.6 ± 1.6%IA/g) [154].

In a following study, further modifications were made in the non-silylated JMV6660 and its position 13 silylated counterpart JMV6659, including the introduction of different linkers and/or the reduction of the Lys^7^–Lys^8^ peptide bond (Table 5) [155]. The affinities of the Ga-tagged silylated analogs were found in the single-digit nM range. Interestingly, JMV7490 (DOTA-*β-*Ala-*ε-*Lys-Lys-Ψ(CH_2_NH)-Lys-Arg-Pro-Tyr-Ile-TMSAla-OH) tagged with Ga displayed the lowest affinity for the NTS_1_R (14.6 ± 4.3 nM), which deteriorated even further when tagged with In (77.5 ± 14.1 nM) [155]. This finding is indicative of the impact of local charge changes on receptor affinity, caused by the different coordination modes of the two metals to DOTA [12,13,14,136]. The stability of all analogs in plasma incubates was found to be enhanced (t_1/2_ ≥ 10 min) compared to the [^68^Ga]Ga-JMV6660 reference (t_1/2_ < 2 min), but the stability of the two TMSAla-OH^13^-modified analogs with the -*β-*Ala-*β-*Ala- linker ([^68^Ga]Ga-JMV7222 and [^68^Ga]Ga-JMV7258; Table 5) was significantly higher (t_1/2_ > 45 min), revealing a synergetic effect of C-terminal silylation and linker type on stability.

The most stable analog turned out to be [^68^Ga]Ga-JMV7490 (Table 5), remaining in vitro intact during the time of the study (45 min). Similarly, [^111^In]In-JMV7490 remained 76% intact in plasma incubates up to 2 h. However, only ≈40% intact [^111^In]In-JMV7490 was found in mice blood collected 5 min pi [155], hinting at the action of NEP, and maybe other proteases not present in the plasma, in its rapid in vivo degradation [82,83,121,122]. In mice bearing HT-29 xenografts, [^68^Ga]Ga-JMV7258 displayed low tumor uptake at 2 h pi (1.5 ± 0.4%IA/g vs. 1.1 ± 0.3%IA/g in block) and unfavorably high kidney uptake (33.6 ± 14.7%IA/g); these results were later verified during a PET imaging experiment [155]. Again, one may suspect a discrepancy between in vitro and the non-evaluated in vivo stability, which implicates NEP (and potentially of other proteases), as a rational explanation for these disappointing results [82,83,121]. Nevertheless, the [^111^In]In-JMV7490 remaining >40% intact in mice circulation at 5 min pi was able to achieve the clear NTS_1_R-targeting of the HT-29 tumor in the same model at 4 h pi (3.6 ± 0.3%IA/g vs. 0.7 ± 0.02%IA/g in a NTS_1_R-negative implant) in support of this assumption. However, kidney values were disadvantageously high at 4 h pi (77.1 ± 8.0%IA/g) [155].

### 5.8. Carbamoylation of a-Methylated Arg^8^

In an interesting approach, a large library of NT(8–13) analogs substituted at positions 11, 12 or 13 by different commercially available amino acid mimics were methylated at the *α*-amine of Arg^8^; the chelator DOTA was coupled via a variety of linkers (with a few thereof containing a fluorinated biphenyl or benzoyl moiety) to the *N^ω^*-carbamoylated Arg^8^ to allow for labeling with the PET radionuclide Ga-68 [156]. Four of these analogs (Figure 12) showed similar NTS_1_R affinity (≈8 nM), and their Ga-68-labeled versions displayed good metabolic stability in serum [156].

Specifically, these four candidates were [^68^Ga]Ga**-21** (Tle^12^; [^68^Ga]Ga**-**DOTA-Lys), [^68^Ga]Ga**-33** (Tle^12^; [^68^Ga]Ga**-**DOTA-Lys-(4-(4-fluorophenyl)Phe-PEG2), [^68^Ga]Ga**-37** (Tle^12^; [^68^Ga]Ga**-**DOTA-(4-(4-fluorophenyl)Phe-PEG2), and [^68^Ga]Ga**-56** (or [^68^Ga]Ga**-**UR-LS130: *β*,*β*-Dmt^11^ (*β*,*β*-dimethyl-Tyr) and [^68^Ga]Ga**-**DOTA-Lys), as depicted in Figure 12. In vivo stability studies reveal that only [^68^Ga]Ga**-56** was excreted >90% intact in the urine up to 45 min pi, in contrast to the other three analogs, which were excreted <6% intact in mice urine. Furthermore, [^68^Ga]Ga**-56** remained practically intact in mice circulation up to 10 min pi. These results emphasize once again the importance of in vivo studies to determine the actual metabolic stability of peptide radioligands, as detailed in the previous chapters. They also show that high in vivo metabolic stability could be achieved by an Ile^12^-analog, which underwent the replacement of Tyr^11^ by *β*,*β*-Dmt^11^, while preserving high-affinity binding to NTS_1_R. A preliminary comparison of the biodistribution patterns of [^68^Ga]Ga**-21/33/37/56** in mice bearing HT-29 xenografts at 45 min pi revealed that the introduction of the lipophilic fluorinated biphenyl moiety in [^68^Ga]Ga**-33** and [^68^Ga]Ga**-37** did not lead to increased uptake in the tumor, as was initially anticipated. The lipophilicity enhancement due to the biphenyl residue was expected to prolong the time of [^68^Ga]Ga**-33** and [^68^Ga]Ga**-37** in blood circulation, thereby boosting tumor uptake. Instead, an unfavorable shift toward hepatobiliary excretion occurred for the two lipophilic [^68^Ga]Ga**-33** and [^68^Ga]Ga**-37**. Eventually, a more detailed biodistribution study was conducted for [^68^Ga]Ga**-56**, including the 10, 25 and 45 min pi time intervals and an extra block group of animals at 45 min pi. Despite the increase in tumor uptake impressively reaching 8.4 ± 2.9%IA/g (vs. 0.94 *±* 0.55%IA/g in block) at 45 min pi and a fast clearance from most physiological tissues, kidney uptake remained disappointingly high (55 *±* 28%IA/g) [156].

### 5.9. The Chelator Placed at the ε-NH_2_ of Lys^6^ of NT(6–13)

In an interesting approach, a series of NT(6–13) analogs, acetylated at the *a*-NH_2_ of N-terminal Lys^6^, carried the chelator of choice (DTPA, DOTA or NOTA) directly or via a linker at its *ε-*NH_2_, as depicted in the upper panel of Table 6. Most of these analogs were Tle^12^-substituted or/and methylated in the Pro^7^-Arg^8^ bond (Table 6) [150,157,158,159,160]. In a similar way, DTPA was coupled at the *ε-*NH_2_ of Lys^6^ in the full 13-peptide chain of native NT yielding [Lys^6^(DTPA)]-NT (Table 6) [150]. In vitro studies revealed that the NTS_1_R affinity was only moderately impaired by the positioning of the [^111/nat^In]In-DTPA at the lateral chain of Lys^6^ in [^111/nat^In]In-NT-20.1 (Ac-Lys(DTPA)-Pro-Arg-Arg-Pro-Tyr-Ile-Leu-OH), [^111/nat^In]In-NT-20.2 (Ac-Lys(DTPA)-Pro-Arg-Arg-Pro-Tyr-Tle-Leu-OH) and [^111/nat^In]In-NT-20.3 (Ac-Lys(DTPA)-Pro-(NCH_3_)Arg-Arg-Pro-Tyr-Tle-Leu-OH; Table 6), which remained in the 0.5–2.5 nM range. Likewise, the NTS_1_R-affinity of [Lys^6^([^111/nat^In]In-DTPA)]-NT was also found within this range (1.8 ± 0.4 nM) [150]. Concerning metabolic stability, non-stabilized [^111^In]In-NT-20.1 was found <1% intact in mouse circulation at 15 min pi, which is less stable even than [Lys^6^([^111^In]In-DTPA)]-NT (≈4% intact). The Tle^12^-substituted [^111^In]In-NT-20.2 was a bit more stable (≈10% intact), whereas double-stabilized (methylation of the Pro^7^-Arg^8^ bond and Ile^12^→Tle^12^-substitution; Table 6) [^111^In]In-NT-20.3 was the most robust analog in the series (>25% intact) [150]. In mice bearing HT-29 xenografts, only [^111^In]In-NT-20.3 displayed clear and NTS_1_R-mediated uptake in the implants at 3 h pi (2.4 ± 0.2%IA/g vs. <0.2%IA/g in block), although this was still lower than kidney uptake (4.8 ± 0.6%IA/g) [150].

These results have prompted the development of related NT-20.3 mimics carrying instead the chelator DOTA at the *ε-*NH_2_ of Lys^6^ to allow for labeling with therapeutic radiometals, such as Y-90 and Lu-177. As shown in Table 6, these were DOTA-NT-20.3 per se, and its two derivatives DOTA-NT-20.4 (methylation of the Arg^8^-Arg^9^ bond instead of the Pro^7^-Arg^8^ bond) and DOTA-LB119 (DOTA coupled to the *ε-*NH_2_ of Lys^6^ via an Ahx linker) [157]. The replacement of DTPA by DOTA had no effect on the NTS_1_R affinity of [^111^In]In-DOTA-NT-20.3, while [^111^In]In-DOTA-LB119 displayed the same affinity for the NTS_1_R (in the 14–16 nM range). However, [^111^In]In-DOTA-NT-20.4 showed a drastic drop of NTS_1_R-affinity (>190 nM). After switching from In-111 to Ga-68 in [^68/nat^Ga]Ga-DOTA-NT-20.3, no effect was observed on NTS_1_R-affinity, whereas switching to Y-90 caused a significant increase in the affinity of [^90/nat^Y]Y-DOTA-NT-20.3 to the NTS_1_R. In the case of DOTA-LB119, switching from In-111 to either Ga-68 or Y-90 equally enhanced NTS_1_R-affinity [157]. These findings underscore the impact of (radio)metal chelate type and position in the molecule, which may have a capacity to interact with its receptor, as often pointed out in this review. With regard to in vivo stability, none of the above three [^111^In]In-radioligands was found intact in mouse circulation at 15 min pi, with [^111^In]In-DOTA-LB119 turning out to be significantly less stable (≈15% intact) than the other two analogs (20–26% intact) [157]. In mice bearing HT-29 xenografts, [^111^In]In-DOTA-NT-20.3 combining high NTS_1_R-affinity and the best in vivo stability outperformed the other members of the series as far as tumor uptake and retention is concerned (2.5 ± 0.2%IA/g at 3 h pi to 1.3 ± 0.2%IA/g at 24 h pi), showing slightly better uptake than its [^111^In]In-DTPA-NT-20.3 congener [157]. The tumor uptake of [^68^Ga]Ga-DOTA-NT-20.3 and [^68^Ga]Ga-DOTA-LB119 was next compared in a PET study, with [^68^Ga]Ga-DOTA-NT-20.3 showing higher tumor uptake and allowing the visualization of very small tumors with PET [157].

Based on these positive findings, [^68^Ga]Ga-DOTA-NT-20.3 was subsequently evaluated in a pancreatic cancer model in mice. Specifically, its diagnostic value in discriminating PDAC from pancreatitis with PET was explored in mice bearing human NTS_1_R-expressing AsPC-1 xenografts or orthotopic tumors and animals with caerulein-induced pancreatitis (along with proper controls), and compared with [^18^F]FDG [158]. The results show that [^68^Ga]Ga-DOTA-NT-20.3 was able to detect cancer lesions, both xenografted and orthotopic. Notably and unlike [^18^F]FDG, [^68^Ga]Ga-DOTA-NT-20.3 was able to clearly discriminate pancreatic cancer (with high NTS_1_R-expession levels) from pancreatitis in the animals, thereby showing valid prospects for clinical translation in PDAC patients [158]. Based on these findings, a first-in-human study was next conducted in three PDAC patients applying PET/CT to evaluate the safety and tolerability of [^68^Ga]Ga-DOTA-NT-20.3 for human use as a new diagnostic tool [159]. The tracer was indeed well tolerated by the patients, showing low background uptake except for in the kidneys, but unexpected uptake in the skeleton not previously observed in the animal models [157,158,159]. Some uptake was evident in the primary lesions and the liver metastases in the three patients, but these results require more rigorous support by studies in more patients with PDAC and other NTS_1_R-expressing malignancies to reveal the true diagnostic prospects of [^68^Ga]Ga-DOTA-NT-20.3 in the clinic [159].

The chelator NOTA was next coupled to the *ε-*NH_2_ of Lys^6^ to enable labeling with other important PET radiometals besides Ga-68, such as the longer-lived Cu-64 (t_1/2_ 12.7 h) and Co-55 (t_1/2_ 17.5 h), allowing for imaging at later time points. This option offers potential advantages in imaging quality due to the higher contrast achieved at later time points against a clearer background. Furthermore, findings are more relevant in a radiotheranostic setting, where the respective therapeutic radiometals (Cu-67 and Co-58m) are employed, because they allow for more accurate dosimetry calculations [160]. A side-by-side preclinical comparison of the three [^68^Ga]Ga-/[^64^Cu]Cu-/[^55^Co]Co-labeled NOTA-NT-20.3 radioligands was conducted in HT-29 cells and HT-29 tumor-bearing mice. In the cell studies, both [^64^Cu]Cu-NOTA-NT-20.3 and [^55^Co]Co-NOTA-NT-20.3 displayed significantly higher cell uptake vs. [^68^Ga]Ga-NOTA-NT-20.3 after 1 h incubation, with [^55^Co]Co-NOTA-NT-20.3 achieving the overall highest cell uptake values. In the animal studies, only [^64^Cu]Cu-/[^55^Co]Co-NOTA-NT-20.3 advantageously allowed for imaging at 24 h pi. Notably, [^55^Co]Co-NOTA-NT-20.3 outperformed [^64^Cu]Cu-NOTA-NT-20.3 by displaying higher tumor uptake combined with a faster hepatic clearance compared with the radiocopper analog [160].

Based on these promising results, a therapeutic study was conducted next with the theranostic [^55/58m^Co]Co-NOTA-NT-20.3 pair. Cobalt-58m (^58m^Co, t_1/2_ = 9.1 h) emits low-range, high-linear-energy-transfer (LET) Auger electrons potentially associated with less radiotoxicities compared with the higher energy electron-emitters often employed in targeted radiotherapy [161,162]. In vitro, [^58m^Co]Co-NOTA-NT-20.3 displayed high cytotoxicity to HT-29 cells. Three groups of mice were next included in the pilot therapy study with [^58m^Co]Co-NOTA-NT-20.3, a week after HT-29 tumor induction, as follows: first—110 ± 15 MBq; second—26 ± 6 MBq; third—PBS [161]. A modest therapeutic response was observed only in the 110 MBq group, while no sign of toxicity was evident in any of the animals. Higher time-integrated activity and/or multiple dosing regimens may be necessary for [^58m^Co]Co-NOTA-NT-20.3 to achieve meaningful therapeutic responses [161].

### 5.10. Branched Analogs

The preclinical evaluation of the branched DT9-DT12 group of analogs labeled with the SPECT radionuclide Tc-99m (Table 2) [118,122] revealed [^99m^Tc]Tc-DT11 as the radioligand of choice for NTS_1_R-positive tumor targeting. In mice bearing human pancreatic cancer AsPC-1 xenografts (Section 4.2.4. in this review), it displayed high tumor uptake as a result of its higher stability and internalization efficacy combined with attractive pharmacokinetics and tumor-to-kidney values >1 [122]. Based on this outcome, DT11 underwent “reshaping” to DT14D after the replacement of the N_4_-chelator at the *α*-NH_2_ of Lys^7^ by DOTA via a (*β*-Ala)_3_ spacer [163]. The spacer was considered necessary in view of previous reports on the negative impact of polar/negatively charged entities at the N-terminus of the NT(8–13) fragment on its interaction capabilities with the NTS_1_R. The replacement of the open chain tetraamine by DOTA allows for labeling with other theranostic radiometals beyond Tc-99m. The new analog DT14D (Figure 13) was next labeled with Ga-67 (as a surrogate for the PET radiometal Ga-68), In-111 (SPECT) and Lu-177 (radiotherapy), and the properties of resulting radioligands were studied and compared in pancreatic AsPC-1 cells and animal models [163].

The three tested radioligands, [^67^Ga]Ga/[^111^In]In/[^177^Lu]Lu-DT14D, displayed high affinity for the human NTS_1_R and internalization in AsPC-1 cells. In vivo they remained >70% intact 5 min after entering mice circulation, showing higher stability than their [^99m^Tc]Tc-DT11 parent [122,163]. They displayed comparable uptake in the AsPC-1 xenografts in mice (3.7–4.4%IA/g) at 4 h pi, which were consistently higher than renal uptake (tumor-to-kidney ratios > 1) [163]. Treatment of the animals with Entresto^®^ in the case of [^111^In]In-DT14D boosted tumor uptake (to ≈8.5%IA/g), but inadvertently led to increases in background levels. The latter were tentatively attributed to the longer residence time of the radioligand in mouse circulation. [^111^In]In/[^177^Lu]Lu-DT14D showed good retention and tumor-to-background contrast at the 24 h pi interval. More importantly, the uptake of [^177^Lu]Lu-DT14D in the xenografts only marginally declined between 24 and 72 h pi (1.8 ± 0.1%IA/g and 1.3 ± 0.2%IA/g, respectively) [163]. These results are very promising for planning the evaluation of DT14D labeled with PET, SPECT or therapeutic radiometals as a new radiotheranostic tool for human NTS_1_R-expressing cancer, without the need for in situ stabilization.

Interestingly, a quite similar approach has been followed concurrently for DOTA-NT-20.3. In this case, two different ABEs have been attached at the *α*-NH_2_ of Lys^6^ instead of the acetyl group, namely, IPBA (4-(*p*-iodophenyl)-butyric acid) [164] or Ibu (2-(4-isobutylphenyl) propionic acid) [165] (Figure 13). The two ABEs have been coupled directly on Lys^6^ without introducing a linker. This may affect the overall in vivo stability of the resulting radiotracers, as previously shown for [^99m^Tc]Tc-DT10–[^99m^Tc]Tc-DT12 (Table 2), whereby resistance to proteolytic enzymes was found to be dependent both on binding to albumin and steric factors [122]. However, an in vivo stability study was conducted neither for [^68^Ga]Ga-DOTA-NT-20.3-IPBA nor for [^68^Ga]Ga-DOTA-NT-20.3-Ibu [164,165]. Both radioligands retained fair affinity to the NTS_1_R, as predicted by dedicated molecular-docking experiments, and were taken up by HT-29 cells via a NTS_1_R-mediated process; they both displayed excellent in vitro stability in mouse serum incubates [164,165], implying resistance to ACE [82,83]. In mice bearing HT-29 xenografts, both radioligands displayed elevated uptake compared to [^68^Ga]Ga-DOTA-NT-20.3, with [^68^Ga]Ga-DOTA-NT-20.3-Ibu outperforming the other two analogs. So, at 2 h pi, the rank of analogs in HT-29 tumor uptake was as follows: for [^68^Ga]Ga-DOTA-NT-20.3-Ibu, 4.7 ± 1.7%IA/g >> [^68^Ga]Ga-DOTA-NT-20.3-IPBA, 2.1 ± 0.2%IA/g > [^68^Ga]Ga-DOTA-NT-20.3, 1.1 ± 0.2%IA/g [164,165]. In all cases, kidney uptake surpassed tumor uptake. The introduction of either ABE had a favorable impact on tumor uptake and pharmacokinetics, by prolonging the systemic circulation and possibly by enhancing the in vivo stability of [^68^Ga]Ga-DOTA-NT-20.3 mimics. However, [^68^Ga]Ga-DOTA-NT-20.3-Ibu achieved better pharmacokinetics over [^68^Ga]Ga-DOTA-NT-20.3-IPBA, not only with regard to tumor uptake but to a clearer background too [165].

Eventually, clinical translation was pursued for both PET tracers in a small number of patients with confirmed lung cancer. Notably, no adverse events were reported and no significant changes were detected in vital signs, laboratory parameters or electrocardiograms after the iv injection of either [^68^Ga]Ga-DOTA-NT-20.3-IPBA or [^68^Ga]Ga-DOTA-NT-20.3-Ibu into the patients enrolled, demonstrating the safety of both radiotracers for human use [164,165]. In the first pilot study with [^68^Ga]Ga-DOTA-NT-20.3-IPBA, three patients with lung adenocarcinoma underwent comparative PET/CT imaging with [^18^F]FDG [164]. [^68^Ga]Ga-DOTA-NT-20.3-IPBA clearly delineated tumor lesions with a tumor-to-lung ratio of 7.9 ± 0.8 at 60 min pi, but [^18^F]F-FDG achieved a higher tumor-to-lung ratio of 11.2 ± 6.3. Furthermore, [^68^Ga]Ga-DOTA-NT-20.3-IPBA showed uptake in several physiological tissues, especially in the kidneys [164]. In the second pilot study, four patients with suspected lung cancer underwent [^68^Ga]Ga-DOTA-NT-20.3-Ibu and paired [^18^F]FDG PET/CT imaging [165]. Lung lesions of all patients, including three cases with lung adenocarcinoma and one case with pulmonary hamartoma, were resected, and the NTS_1_R expression was determined by applying immunohistochemistry (IHC) [165]. [^68^Ga]Ga-DOTA-NT-20.3-Ibu visualized all lung adenocarcinoma lesions, with the uptake found to be dependent on NTS_1_R expression density, whereas the NTS_1_R-negative pulmonary hamartoma was not visualized. Significant activity accumulation was evident in the kidneys and the urinary bladder. Overall, the study showed [^68^Ga]Ga-DOTA-NT-20.3-Ibu to be a safe diagnostic molecular probe, allowing for the accurate, non-invasive assessment of tumor NTS_1_R-expression with PET. Further clinical work is definitely needed to confirm its true diagnostic value or/and its application in a radiotheranostic framework [165].

## 6. Non-Peptidic NTS_1_R-Antagonists in Cancer Radiotheranostics—First Impressions

A recent shift of paradigm in the development of anti-cancer radiotheranostics targeting GPCRs is the introduction of an increasing number of radiolabeled receptor-antagonists [5]. The latter have repeatedly achieved higher tumor targeting in animal models and in patients, combined with a much faster clearance from physiological tissues. Still, the exact mechanisms underlying this behavior have not been adequately elucidated yet [5]. In the case of radiolabeled somatostatin subtype 2 receptor (SST_2_R) antagonists, it was shown that they bind to both active and inactive forms of the SST_2_R on the membrane of cancer cells, thereby increasing their overall cell uptake [166]. Another important advantage of receptor antagonists is their inherent biosafety, based on their inability to activate their cognate receptor upon binding. Thus, they do not elicit acute adverse pharmacological effects after iv injection to patients. A plethora of radiolabeled antagonists based on peptide motifs have broadly shown convincing properties for clinical use [5]. Inconveniently though, peptide motifs for GPCR antagonists are not always available. Instead, small organic molecules have been occasionally used as motifs for the development of radiolabeled GPCR antagonists, with fair success thus far achieved [167,168,169].

A first potent non-peptidic NTS_1_R-antagonist SR 48692 (Figure 14) was introduced in 1993, showing no intrinsic agonist activity, as well as oral bioavailability, a long duration of action, and the inhibition of most of the in vitro and in vivo effects of NT [170]. A few years later, SR 142948A (Figure 14) was introduced as a new and extremely potent NTS_1_R antagonist with a wider spectrum of antagonistic activity than SR 48692 [171].

Subsequently, a series of SR 142948A analogs were developed for labeling with bi/trivalent theranostic radiometals, carrying the DOTA chelator via various linkers (Figure 15) [172,173,174,175].

Compounds BP-227 and BP-228 were first labeled with In-111; their In-tagged versions showed good affinities for the NTS_1_R (5.3 nM and 14.4 nM, respectively), whereas [^nat^In]In-3BP-483, a 3BP-227 mimic with the original adamantane group replaced by cyclohexane, showed significantly impaired affinity for the NTS_1_R (75 nM). Their uptake in HT-29 mice xenografts at 6 h pi followed this affinity trend, with [^111^In]In-BP-227—8.4 ± 3.1%IA/g ≥ [^111^In]In-BP-228—8.0 ± 5.0%IA/g > [^111^In]In-BP-483—1.6 ± 0.5%IA/g. Interestingly, the highest retention was exhibited again by [^111^In]In-BP-227; the tumor uptake values at 24 h pi ended up as [^111^In]In-BP-227—2.3 ± 1.3%IA/g > [^111^In]In-BP-228—0.8 ± 0.2%IA/g ≈ [^111^In]In-BP-483—0.8 ± 0.4%IA/g. It should be noted that clearance from physiological tissues was advantageously much faster than from xenografts. Consequently, tumor-to-tissue ratios remained >1 always for all healthy organs, including tumor-to-kidney ratios [172]. In view of these promising findings, BP-227 was selected for further evaluation as a radiotheranostic agent and labeled with the beta emitter Lu-177. The SPECT/CT imaging of an HT-29 tumor-bearing mouse with [^177^Lu]Lu-BP-227 revealed high-contrast uptake in the xenografts for a period up to 69 h pi (19.0 ± 3.6%IA/g at 3 h pi vs. 2.7 ± 1.6%IA/g at 69 h pi). This set the basis for the therapy study that followed, involving a control mice group, a group receiving 110-MBq and a second animal group receiving 165-MBq [^177^Lu]Lu-BP-227 [173]. The median tumor doubling time in controls (5.5 d) increased to 17.5 d and 41.0 d for the two treated groups, respectively. Moreover, the median relative tumor volume at day 23 after treatment was reduced by 55% and by 88%, respectively, in the two [^177^Lu]Lu-BP-227-treated groups. At the same time, the kidney histology and clinical results were identical between the two radiotherapy groups and controls, revealing no acute renal damage induced by radiotherapy [173].

These exciting results prompted a translational pilot study conducted in six patients with confirmed and very advanced PDAC [174]. Initial SPECT/CT with [^177^Lu]Lu-BP-227 was applied to select candidates for radiotherapy, revealing five out of the six with high uptake in pathological lesions, as shown in the representative SPECT/CT imaging results for two patients in Figure 16 [174].

Treatment with [^177^Lu]Lu-BP-227 with activities of 5.5 and 5.1 GBq was applied to two of the patients (patient 5 and 6) without evident radiotherapeutic effects. One patient (patient 3) with very advanced and spread disease, involving hepatic, pulmonary, and lymph node metastases, infiltration of the visceral blood vessels, and peritoneal carcinomatosis [174] with massive ascites production and poor clinical condition, first received an exploratory iv injection of 1.5 GBq [^177^Lu]Lu-BP-227 to assess tumor uptake and organ dosimetry. Then, the patient received a series of three intraperitoneal administrations of [^177^Lu]Lu-BP-227 (6.4, 7.5, and 5.5 GBq) at 8–10-week intervals. This patient had a partial response showing the significant improvement of symptoms and quality of life, and survived 11 months after the start of [^177^Lu]Lu-3BP-227 therapy. It should be noted that the administration of [^177^Lu]Lu-3BP-227 was well tolerated by all patients, with the kidneys identified as the dose-limiting organ and the most severe adverse event being reversible grade 2 anemia [174]. This important study has provided evidence that NTS_1_R-targeted radiotheranostics of PDAC is indeed a feasible option with the application of suitable receptor antagonists.

Two further DOTA-conjugated SR 142948A analogs were subsequently developed, the triazolyl-linked FAUC 469 and its amide-linked counterpart DOTA-CL 156 (Figure 15) [175]. They were labeled with Lu-177 via studies in AsPC-1 cells and tumor-bearing mice. They both displayed sub-nM affinities for the NTS_1_R and high stability in human plasma. During biodistribution, they achieved outstanding uptake in the AsPC-1 xenografts at 4 h pi ([^177^Lu]Lu-FAUC 469: 22.4 ± 3.3%IA/g; [^177^Lu]Lu-DOTA-CL 156—34.2 ± 2.5%IA/g) against low kidney and liver uptake (e.g., for [^177^Lu]Lu-DOTA-CL 156—0.9 ± 0.5%IA/g and 1.5 ± 0.5%IA/g, respectively) [175]. Radiotherapy with 25 MBq [^177^Lu]Lu-FAUC 469 resulted in marked tumor growth inhibition vs. controls, while renal/liver histology and blood values were normal. These results validate [^177^Lu]Lu-FAUC 469 as a promising radiotheranostic candidate in PDAC, and clinical translation is expected to follow [175].

Based on previous observations on the high stability of Cu-64/67 complexes with NOTA [160], CB (CB-TE2A, 1,4,8,11-tetraazabicyclo [6.6.2]hexadecane-4,11-diacetic acid) [176,177], and DSar (diamine-sarcosine or DiAmSar: 1,8-Diamino-3,6,10,13,16,19-hexaazabicyclo [6,6,6]-eicosane) [178], SR 142948A analogs were synthesized by the coupling of these chelators via different linkers (Figure 17) [176,177,179].

The multi-amine (MA) macrocyclic chelators CB and DSar have alternatively served as linkers, along with linear MAs for coupling the desired macrocyclic chelator (DOTA, NOTA and derivatives thereof) to SR 142948A. A good number of the resultant conjugates were also labeled with Ga-68, as well as with radiocobalt (Co-55/Co-58m) for use in a radiotheranostic framework [176,179].

The introduction of positively charged radiometal chelates or/and polyamine macrocyclic linkers boosted the uptake and the retention of resulting SR 142948A-based radioligands in the NTS_1_R-positive xenografts in the animal models. For example, [^55^Co]Co-NT-CB-NOTA achieved remarkable uptake in the HT-29 tumors in mice at 1 h pi (20.1 ± 2.1%IA/g) combined with excellent retention at 24 h pi (12.5 ± 1.3%IA/g). Most importantly, background radioactivity levels rapidly declined with time, including the kidneys in the same time interval (9.2 ± 1.4%IA/g to 0.2%IA/g), resulting in notably high-contrast imaging at the longer time intervals and paving the way for safe and effective radiotherapy [176].

## 7. Miscellaneous

A number of alternative efforts to bring forward clinically useful NTS_1_R-directed radioligands in anti-cancer strategies will be very briefly reported in this chapter. An important approach toward this goal has involved the development of multimeric peptides, both homo- and heteromeric. This route is expected to increase target cell avidity, in vivo metabolic stability, and overall tumor-targeting efficacy. As a result, higher diagnostic sensitivity and possibly better therapeutic options may be reached. All the same, certain inherent flaws to this concept are becoming apparent, such as for example the longer retention of the resultant constructs in physiological tissues (including the liver and the kidneys), which eventually have a negative impact on pharmacokinetics [20,53,180,181,182,183,184]. The simultaneous targeting of two GPCRs co-expressed in cancer lesions can be alternatively achieved by the use of radioligand cocktails [183]. This approach was recently explored in the gastrin-releasing peptide receptor (GRPR) and NTS_1_R-positive prostate cancer after the administration of a cocktail of a radiolabeled GRPR-antagonist and an NTS_1_R-radioligand [185].

Several other studies have brought to light radioligands with moderate to significant success in targeting NTS_1_R cancers beyond PDAC or colon adenocarcinoma, which were the focus of the present review. Thus, preclinical results with NTS_1_R radioligands in the respective cell and mice models as well as in patients have already been reported for prostate cancer [45,186,187,188,189,190], lung cancer [30,164,165,191,192], or mammary carcinoma [46,193,194]. This area of research is currently ongoing.

## 8. Conclusions

Based on the information presented in this review, it is evident that the field of NTS_1_R-directed anti-cancer radiotheranostics has been remarkably active and fruitful. A major focus of this effort has been the high density of NTS_1_R expression in PDAC, providing the molecular basis for the urgently needed diagnosis at earlier stages and the application of targeted radionuclide therapy to improve the disappointingly low 5-year survival rate seen today. At the same time, patients with an increasing variety of cancer types are becoming candidates that could benefit from the latest developments in NTS_1_R-directed anti-PDAC agents, including lung cancer, prostate cancer and mammary carcinoma.

The major hurdle in achieving convincing clinical results has undoubtedly been the propensity of NT-based peptide radioligands towards fast proteolytic degradation, which has undermined the supply of intact radioligand molecules to NTS_1_R-expressing pathological lesions. Chemists and pharmacists have shown great ingenuity and zeal in addressing this challenge. As detailed in this review, several chemical interventions were attempted, such as a series of modifications in the C-/N-termini or/and within the peptide chain, branching and coupling to lateral albumin binding or PEG chains, the reduction, methylation or triazolyl-substitution of peptide bonds, multimerization, and so on and so forth. Further approaches have included the application of key protease inhibitors to in situ stabilize radiolabeled analogs. Eventually, most clinical results—wherever available—have turned out to be sub-optimal thus far.

Switching from analogs based on NTS_1_R antagonists instead of agonists has recently provided the first convincing evidence regarding the suitability of the whole NTS_1_R radioligand anti-cancer approach. It appears that chemical modification and metabolic stabilization alone have been insufficient to address the problem of the low NTS_1_R-targeting of pathological lesions in patients. The introduction of NTS_1_R antagonists rather implies a different biochemical setting at play, possibly similar to what we have seen in the case of radiolabeled SST_2_R-antagonists. The latter were shown to bind to more sites on the cell membrane of cancer cells compared with their agonist counterparts. The validity of this or other scenarios remains to be fully explored in the case of NTS_1_R antagonist radioligands. The further evaluation of truly effective analogs in pioneer clinical studies is expected to pave the way for a broader application of radiolabeled NTS_1_R-specific drugs in human cancer, and help in better elucidating the mechanisms underlying its onset and propagation in different phases and phenotypes of the disease.

## Figures and Tables

**Figure 1:**
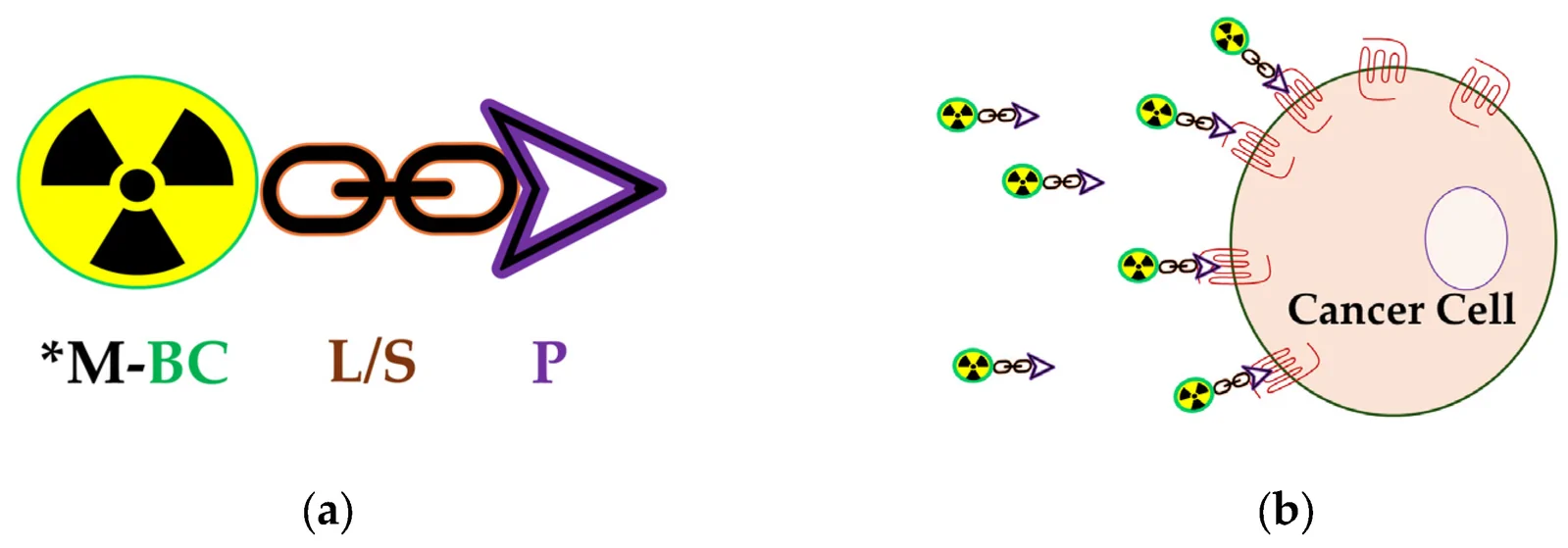
Peptide radioligands and GPCR targets. (**a**) The peptide radioligand comprises the peptide sequence (P), which is the receptor-recognition site, the radiometal (*M) stably bound by the bifunctional chelator (BC) and a linker or spacer (L/S) intercepted between these two parts. (**b**) Peptide radioligand molecules targeting GPCRs on the surface of a cancer cells.

**Figure 2:**
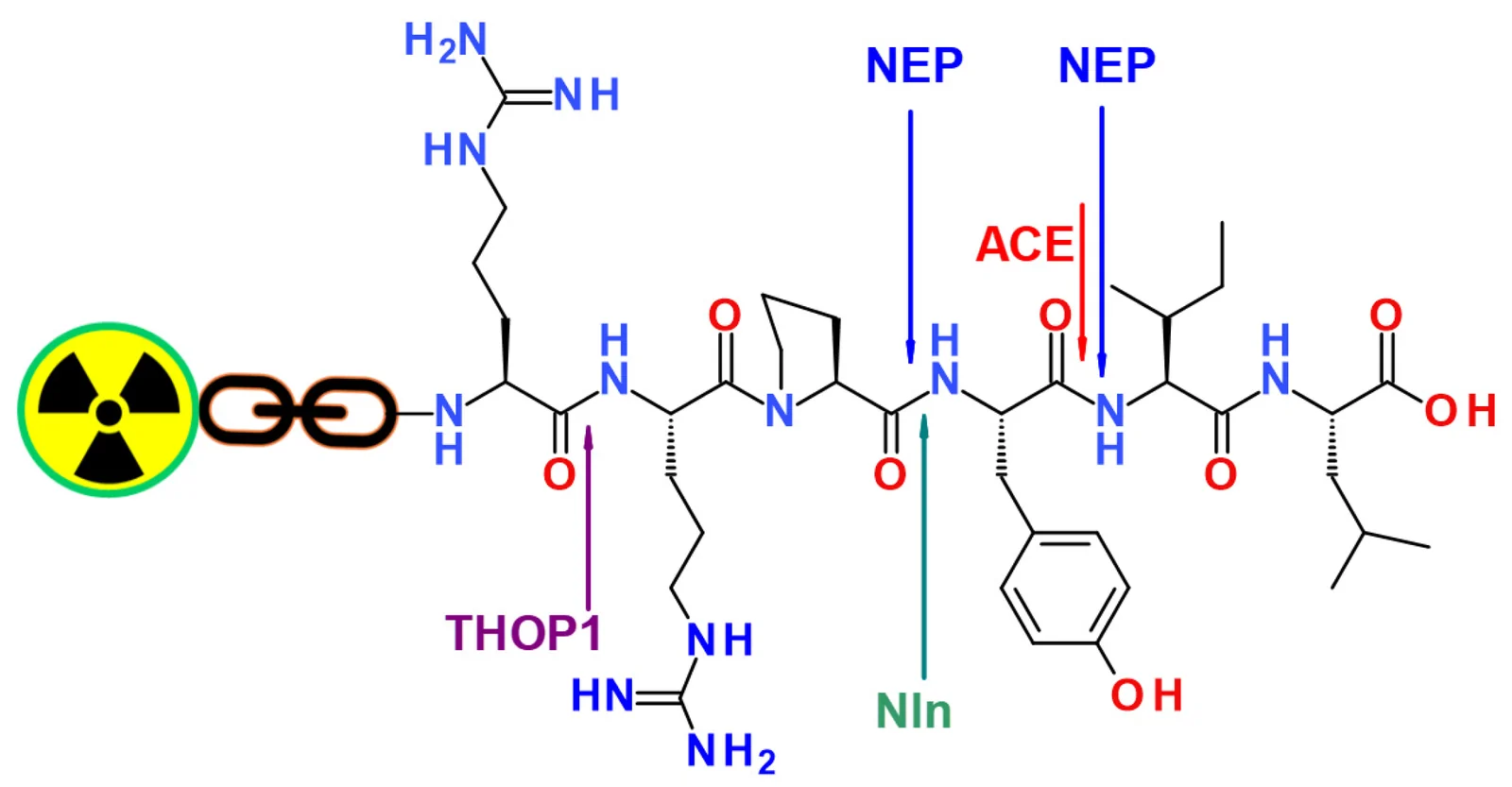
Structure of a hypothetical radioligand, with the radiometal chelate attached via the linker at the *α*-amine of N-terminal Arg of NT(8–13). The arrows indicate the proteases participating in the hydrolysis of the three cleavage sites in the peptide chain. Violet, THOP1: Arg^8^–Arg^8^. Green, Nln: Pro^10^–Tyr^11^. Red, ACE: Tyr^11^–Ile^12^. Blue, NEP Pro^10^–Tyr^11^ and Tyr^11^–Ile^12^.

**Figure 3:**
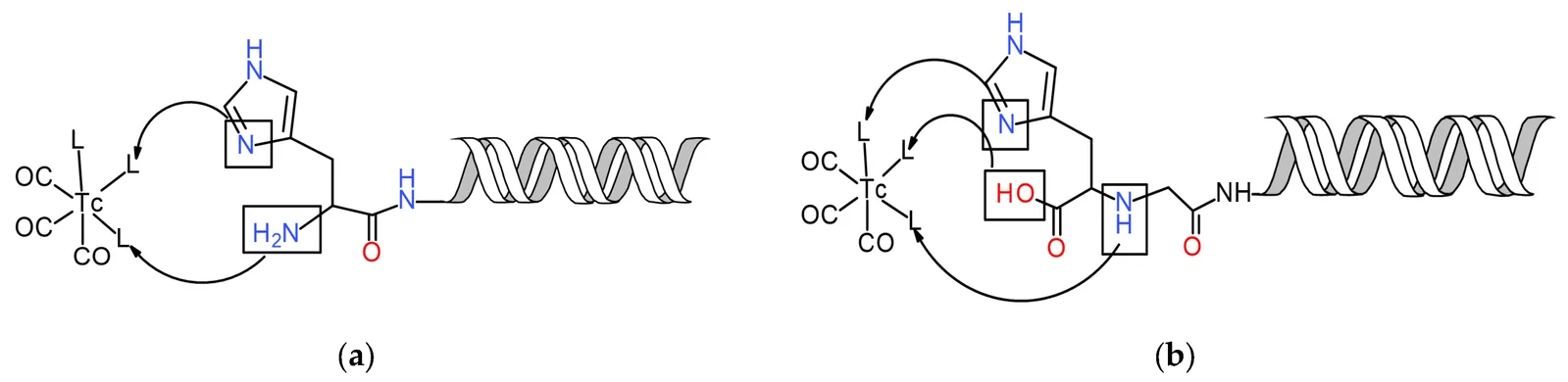
Coordination of the *fac*-[Tc(CO)_3_(L)_3_] synthon (L, e.g., H_2_O) with either (**a**) His or (**b**) *N*^α^-Ac-His proceeds by replacement of either two L monodentate co-ligands by His, acting as a bidentate co-ligand, or all three Ls by *N*^α^-Ac-His, acting as a tridentate co-ligand; coordinating groups are indicated by rectangular frames, while arrows indicate the replacement of L by these donors. His and *N*^α^-Ac-His are attached at the N-terminal amine of a peptide, like NT(8–13) and its analogs, including those N-elongated by amino acid or other types of residues.

**Figure 4:**
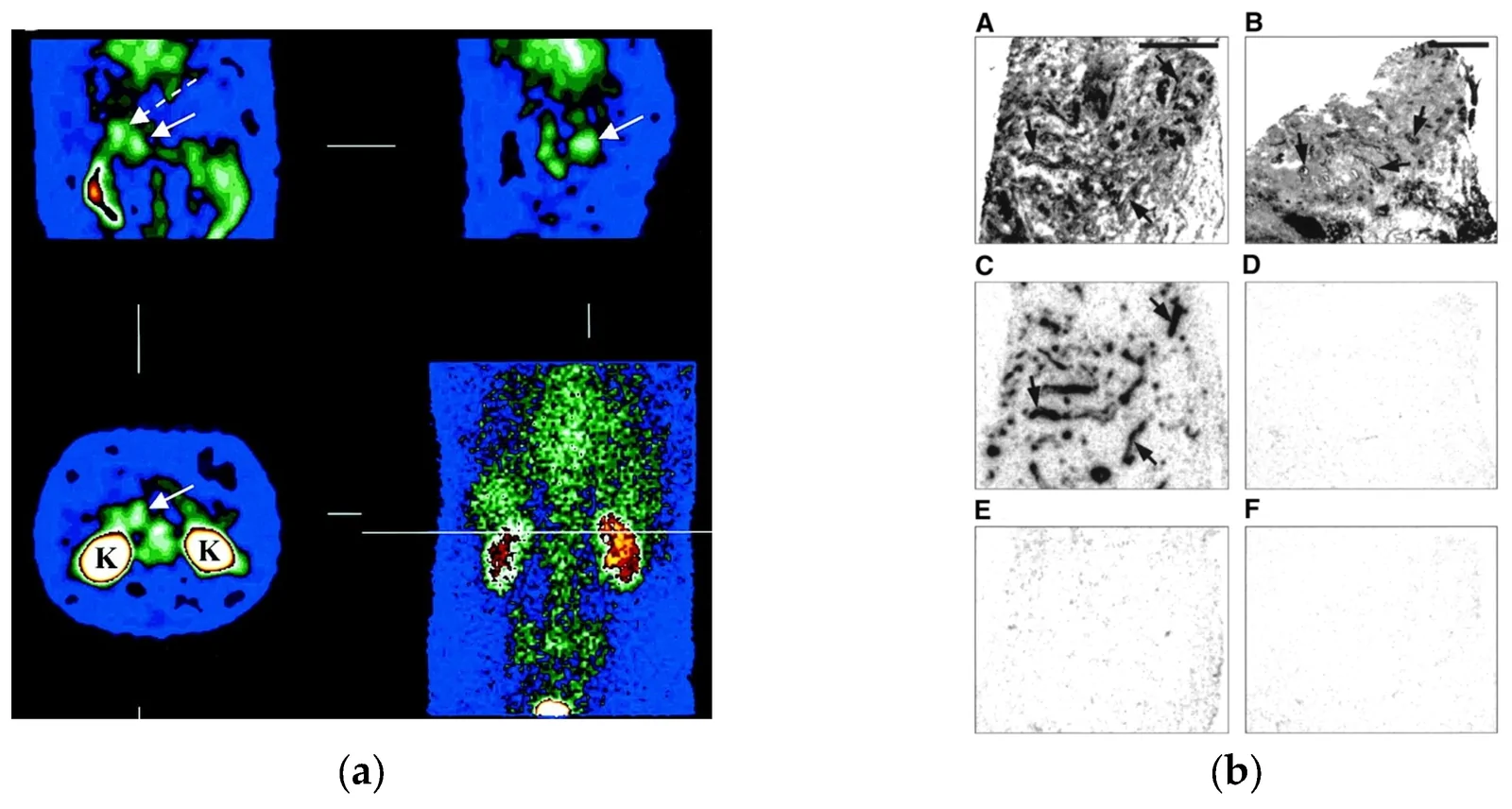
Representative results after injection of [^99m^Tc]Tc-NT-XI in PDAC patients. (**a**) Abdominal tomoscintigraphic sections recorded 3 h after injection of patient 4; (**upper left**) coronal, (**lower left**) transversal, (**upper right**) sagittal and (**lower right**) planar reference scintigraphy; the solid arrow shows pancreatic head tumor; the dashed arrow indicates duodenum. K: kidneys. (**b**) In vitro autoradiographic evaluation of NTS_1_R expression in tumor samples of patients 3 and 4. The tumor of patient 4 (**A**,**C**,**E**) had a very high density of NT receptors, whereas the tumor of patient 3 (**B**,**D**,**F**) was devoid of NT receptors. (**A**,**B**) Sections stained with hematoxylin–eosin. Arrows indicate ductal pancreatic cancer. Bars = 1 mm. (**C**,**D**) Autoradiograms show total binding of [^125^I]I-Tyr^3^-NT. Neoplastic pancreatic ducts (arrows) are strongly labeled in (**C**) but not in (**D**). (**E**,**F**) Autoradiograms show the absence of nonspecific binding in the presence of 10^−6^ mol/L NT. This research was originally published by Buchegger et al. in *J. Nucl. Med.* **2003** *44* (10), 1649–1654 [93].

**Figure 5:**
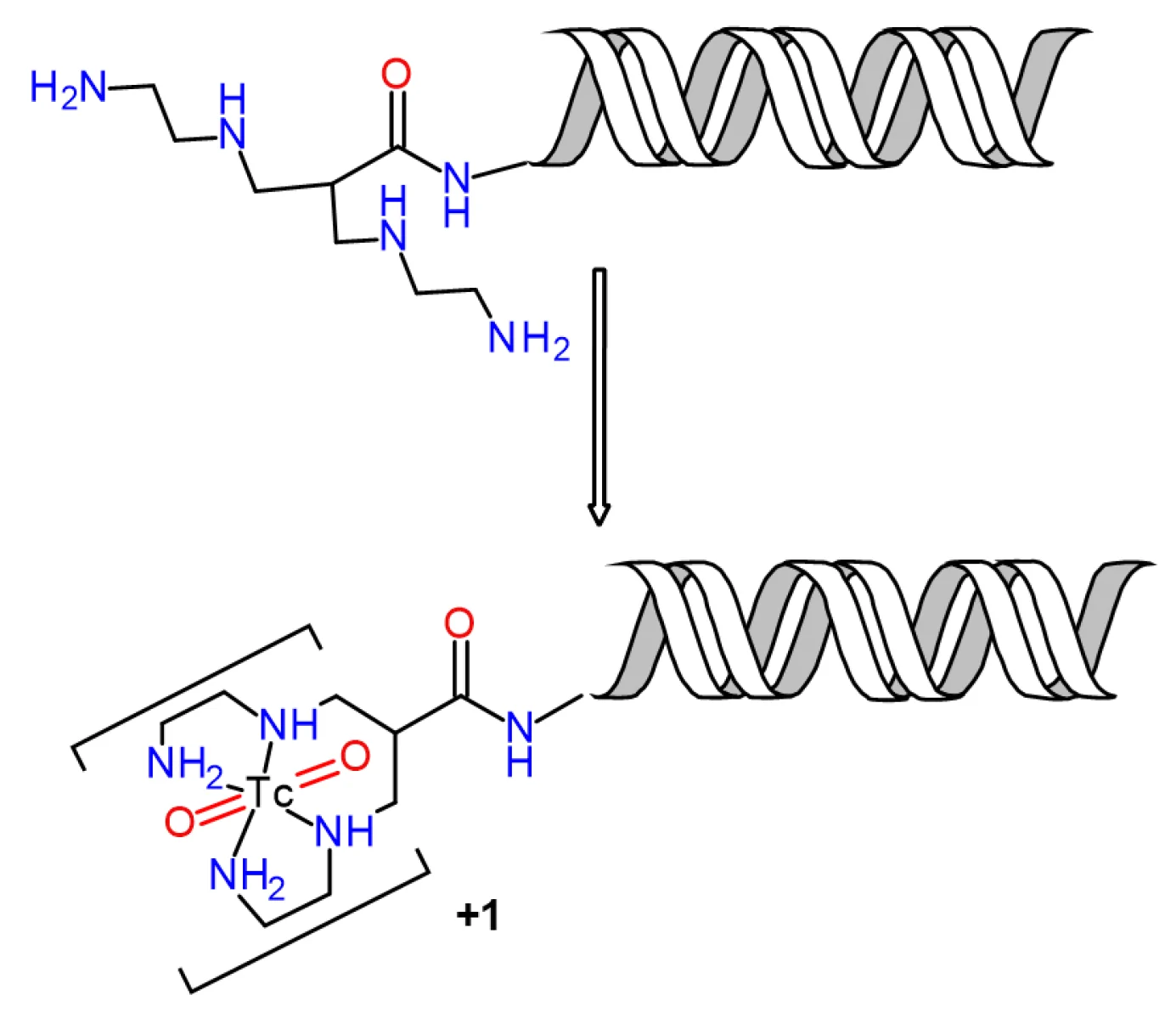
Coordination of bifunctional acyclic tetraamine with Tc-99m; the tetraamine having the four nitrogen atoms at the equatorial plane wraps around the *trans*-[Tc(V)(O)_2_]^+^ core, forming a monocationic octahedral radiometal complex. The tetraamine has been attached to the NT(8–13) analogs via the carboxy functionality at position 6 via a linker.

**Figure 6:**
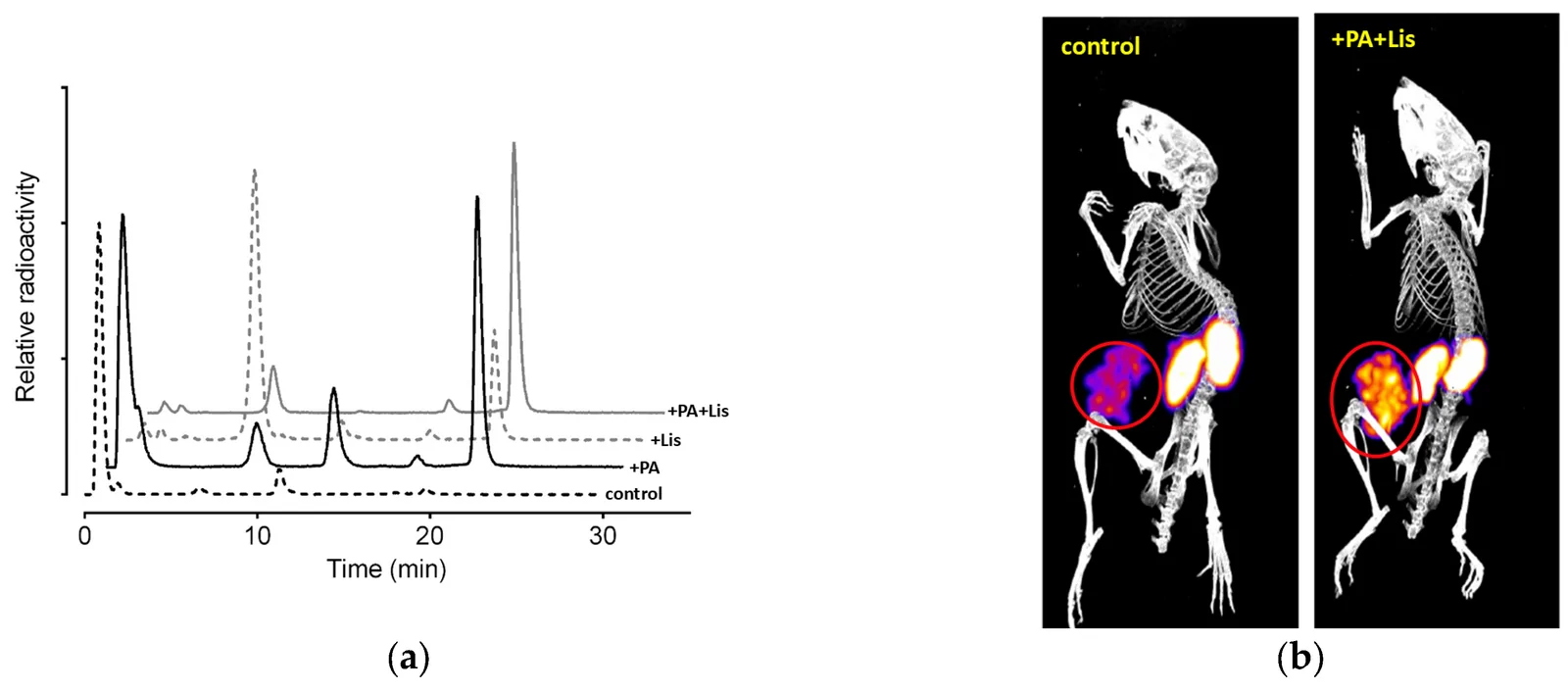
Correlation of [^99m^Tc]Tc-DT1 in vivo stability and the NTS_1_R-targeting of human colon adenocarcinoma WiDr xenografts in mice, revealing the impact of in situ NEP or/and ACE inhibition. (**a**) Analysis of HPLC blood samples collected 5 min pi of [^99m^Tc]Tc-DT1 alone (control), coinjected with PA, or Lis, or both, showing additive stabilization effects during PA+Lis treatment. (**b**) SPECT/CT imaging with [^99m^Tc]Tc-DT1 at 4 h pi, showing significantly improved targeting of the WiDr tumor in the animal coinjected with the PA+Lis combination (**right**) compared with the untreated control (**left**); tumors are highlighted by red circles (adapted from Kanellopoulos et al. *Pharmaceutics* **2020**, *12* (6); *12*, 528. [82]).

**Figure 7:**
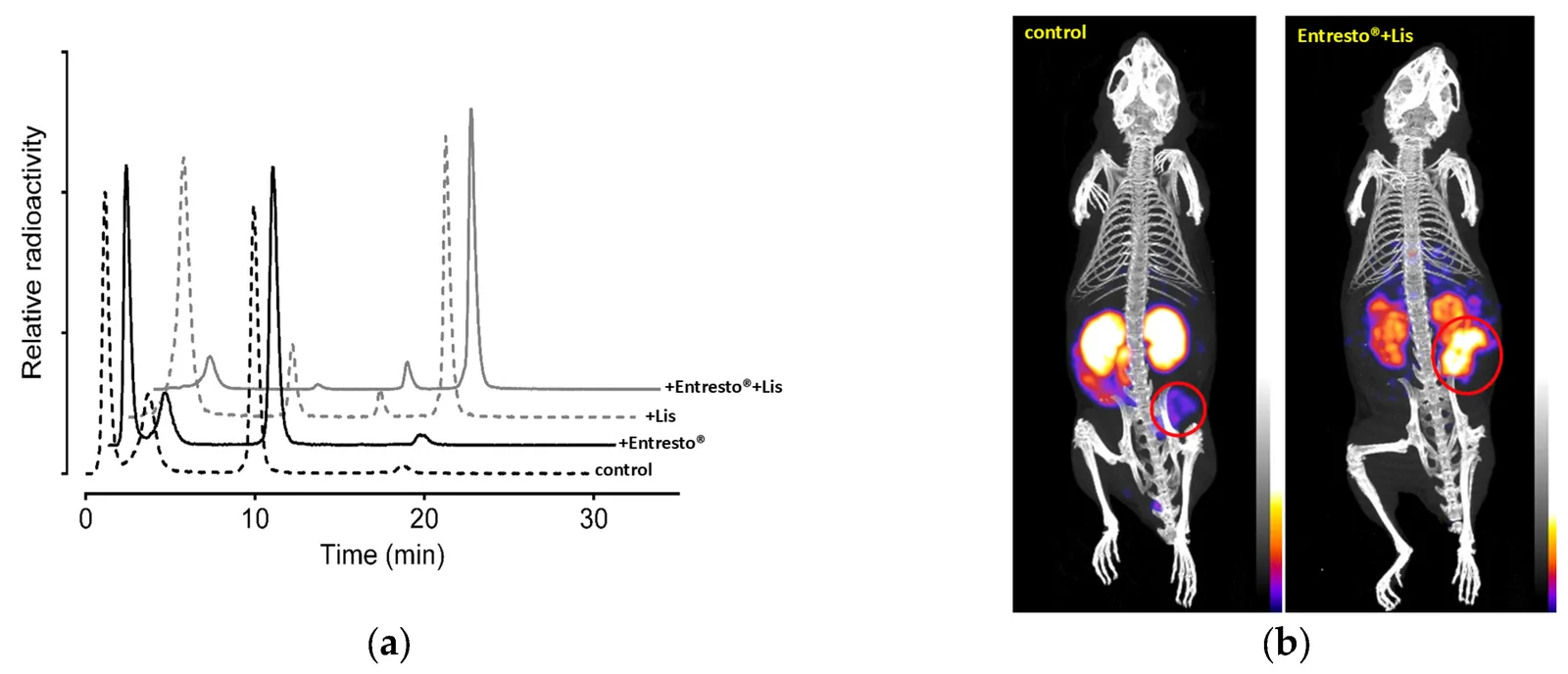
Correlation of [^99m^Tc]Tc-DT1 in vivo stability and NTS_1_R-targeting of human AsPC-1 xenografts in mice, revealing the impact of in situ NEP or/and ACE inhibition. (**a**) Analysis of HPLC blood samples collected 5 min pi of [^99m^Tc]Tc-DT1 in untreated mice (control), or treated with Entresto^®^, or with Lis, or with Entresto^®^+Lis, revealing the highest stabilization effects in the Entresto^®^+Lis group. (**b**) SPECT/CT imaging with [^99m^Tc]Tc-DT1 at 4 h pi, showing significantly improved targeting of the AsPC-1 tumor in the animal treated with Entresto^®^+Lis (**right**) compared with the untreated control (**left**); tumors are highlighted by red circles. (Adapted from Kanellopoulos, et al. *Int. J. Mol. Sci.* **2020**, *21* (21); 7926. [83]).

**Figure 8:**
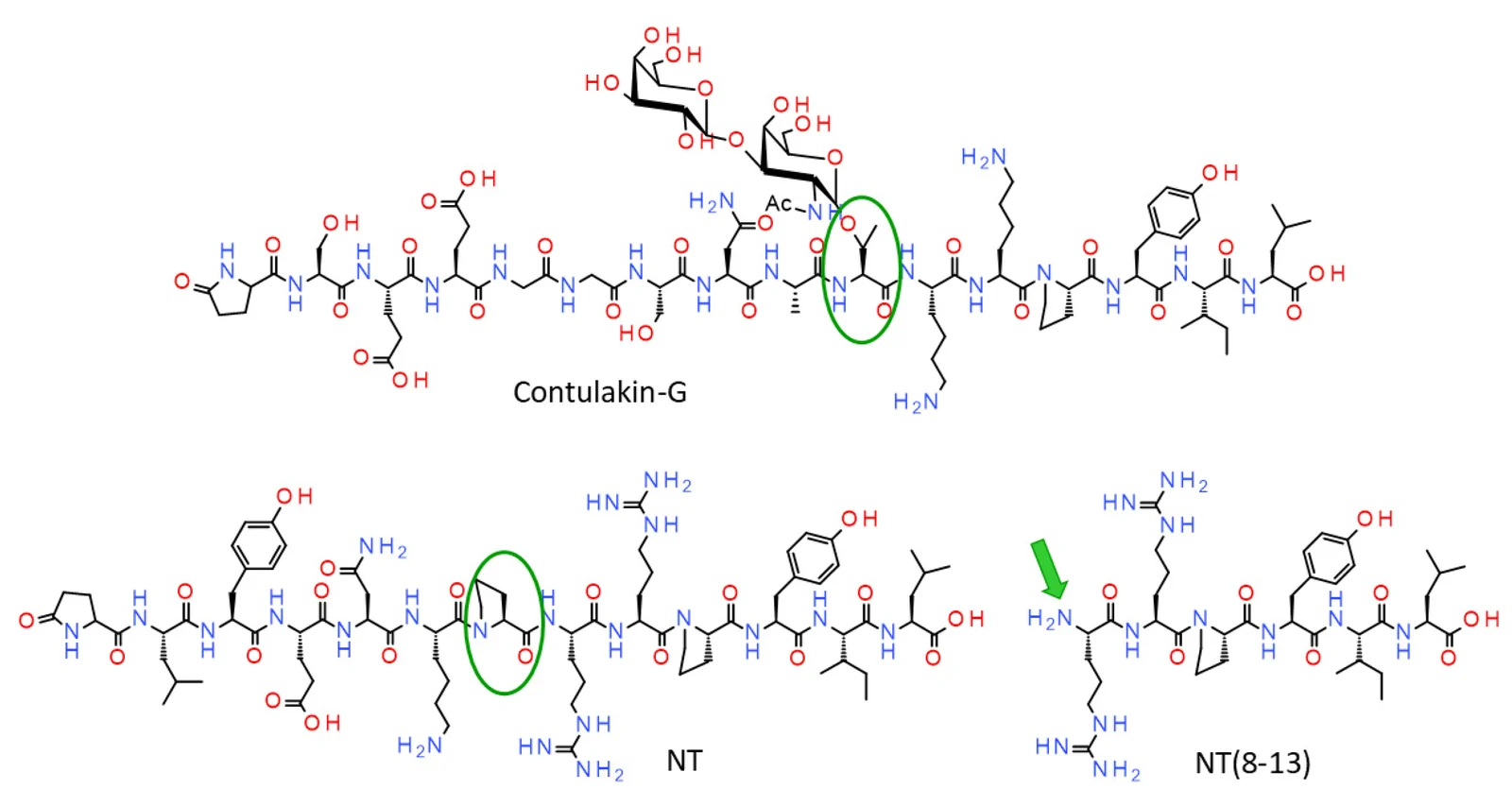
Contulakin-G (upper structure), NT (lower-left structure) and NT(8–13) (lower-right structure); the green circles indicate the positions of the lateral chain attachment (Thr^10^ for contulakin-G and Pro^7^ for NT), tolerated by the NTS_1_R. For the NT(8–13) fragment, N-elongation (green arrow) is required for lateral chain attachment, e.g., by a Lys residue and covalent coupling at its *ε*-amine.

**Figure 9:**
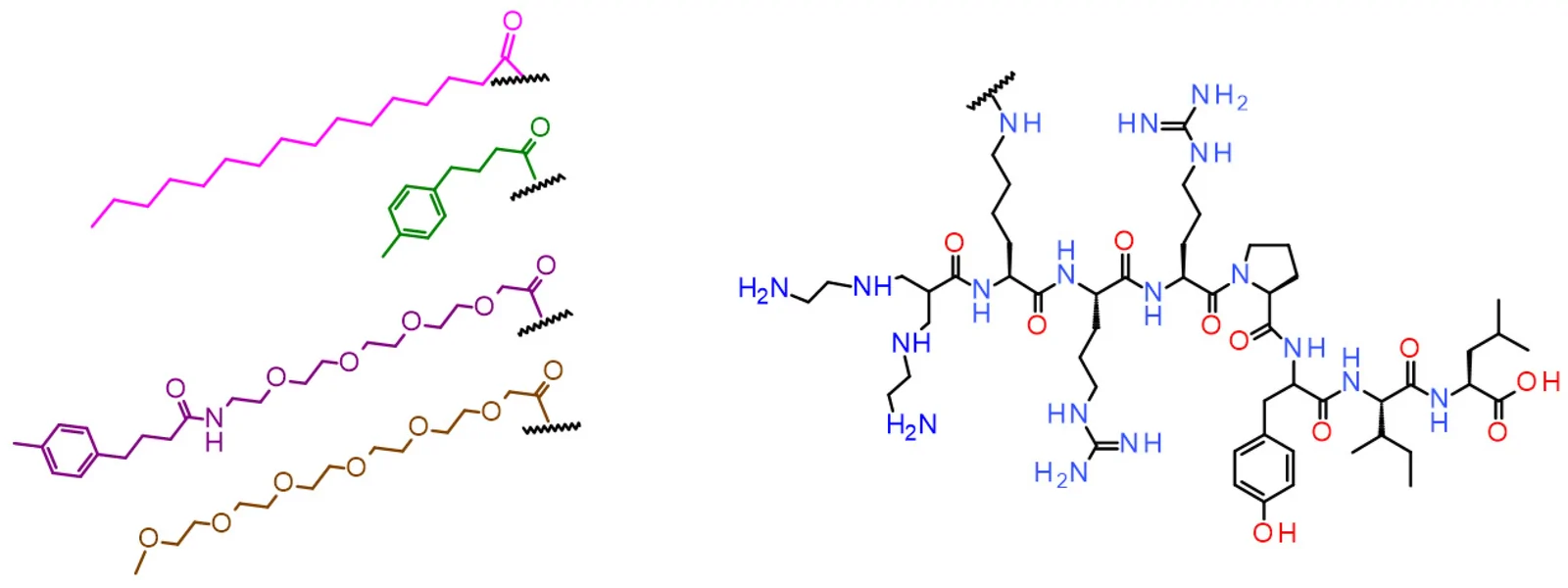
Branched DT analogs with lateral chains attached at the *ε*-NH_2_ of Lys^7^ in the N-elongated NT(8–13) fragment; lateral chains include palmitoyl (pink, DT9), MPBA (green, DT10), MPBA-PEG4 (violet, DT11), or CO-CH_2_-PEG5-OMe (brown, DT12).

**Figure 11:**
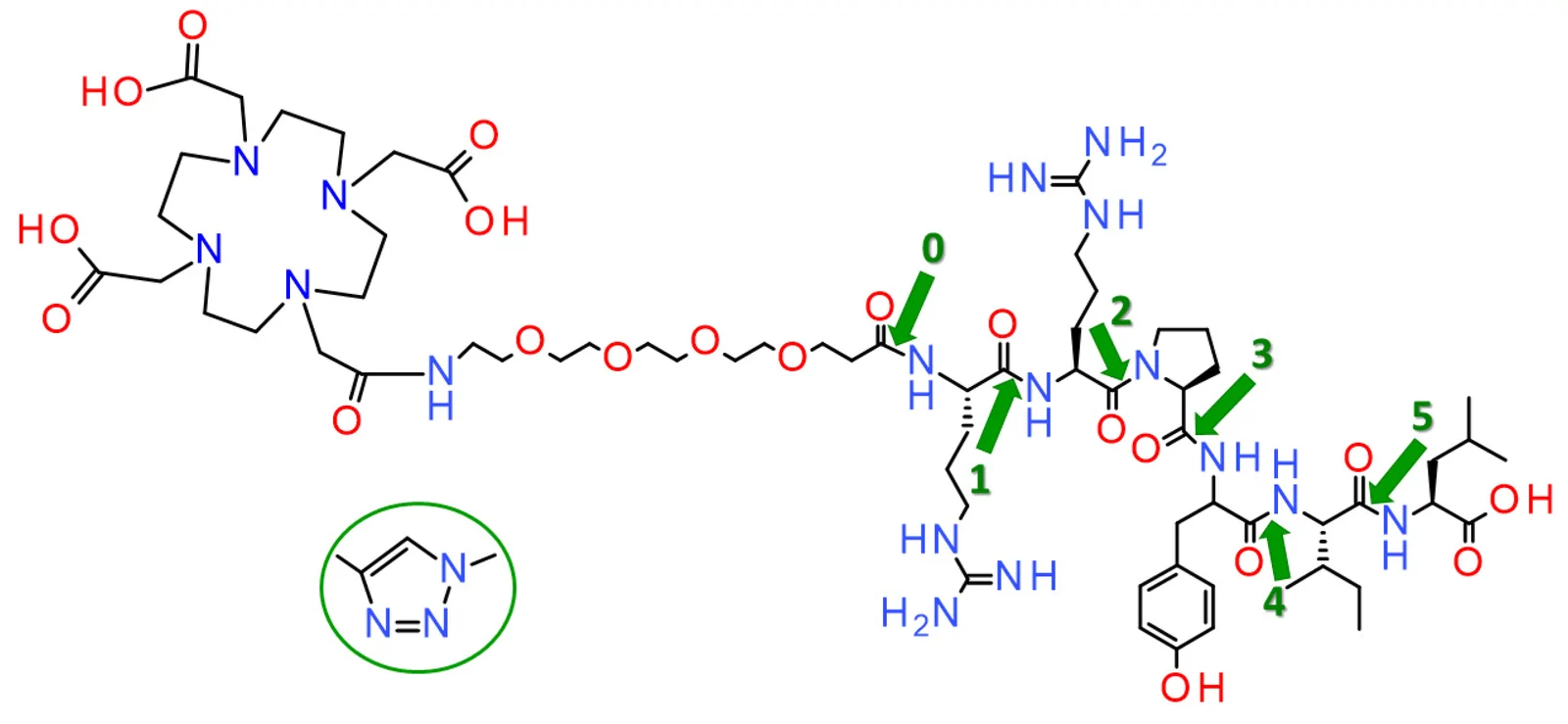
The NT(8–13) fragment coupled to DOTA via the amine-PEG4-propionic acid linker. Green arrows indicate positions of amide bonds, systematically substituted by their isostere 1,2,3-triazoles: 0, PEG4-Arg^8^; 1: Arg^8^-Arg^9^; 2, Arg^9^-Pro^10^; 3, Pro^10^-Tyr^11^; 4, Tyr^11^-Ile^12^ and Ile^12^-Leu^13^; the structure of 1,4-disubstituted 1,2,3-triazole used as an amide bond surrogate is depicted separately in the green circle. In a few of the analogs, Ile^12^ has been replaced by Tle^12^.

**Figure 12:**
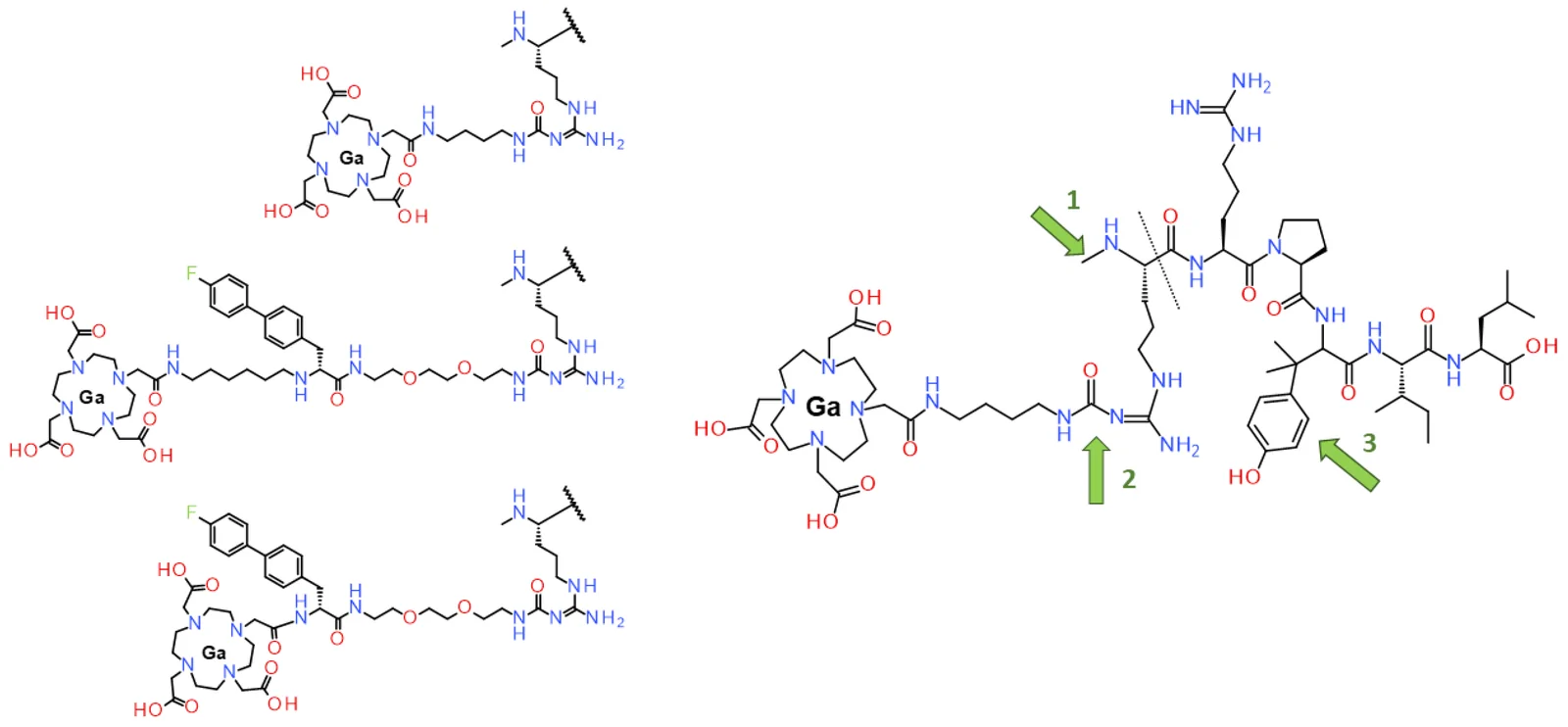
The chelator DOTA coupled to methylated Arg^8^ (arrow 1) of NT(8–13) analogs via *N^ω^*-carbamoylation (arrow 2) in several NT(8–13) analogs labeled with Ga via different linkers. Left part, from top to bottom: [^68^Ga]Ga**-21** (Tle^12^; [^68^Ga]Ga**-**DOTA-Lys), middle [^68^Ga]Ga**-33** (Tle^12^; [^68^Ga]Ga**-**DOTA-Lys-(4-(4-fluorophenyl)Phe-PEG2), and lower [^68^Ga]Ga**-37** (Tle^12^; [^68^Ga]Ga**-**DOTA-(4-(4-fluorophenyl)Phe-PEG2. On the right-hand side, the structure of the best-performing analog [^68^Ga]Ga-**56** ([^68^Ga]Ga-UR-LS130) is shown with *β*,*β*-dimethyl-Tyr (*β*,*β*-Dmt; arrow 3) replacing Tyr^11^.

**Figure 13:**
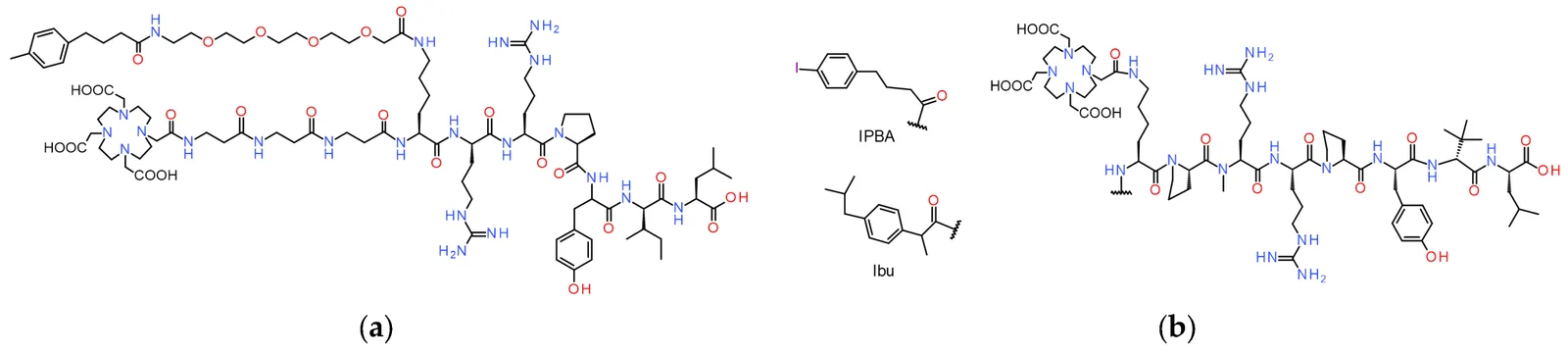
Branched DOTA-NT analogs. (**a**) DOTA is coupled to the *α*-NH_2_ of Lys^7^ of the [Lys^7^]NT(7-13) fragment via a (*β*-Ala)_3_ spacer, while the lateral MPBA-PEG4 chain is attached to its *ε*-NH_2_ yielding DT14D, a DT11 mimic suitable for labeling with theranostic radiometals; (**b**) The NT-20.3 analog has been modified at the *α*-NH_2_ of Lys^6^ by the attachment of either IPBA (4-(*p*-iodophenyl)-butyric acid) or Ibu (2-(4-isobutylphenyl) propionic acid).

**Figure 14:**
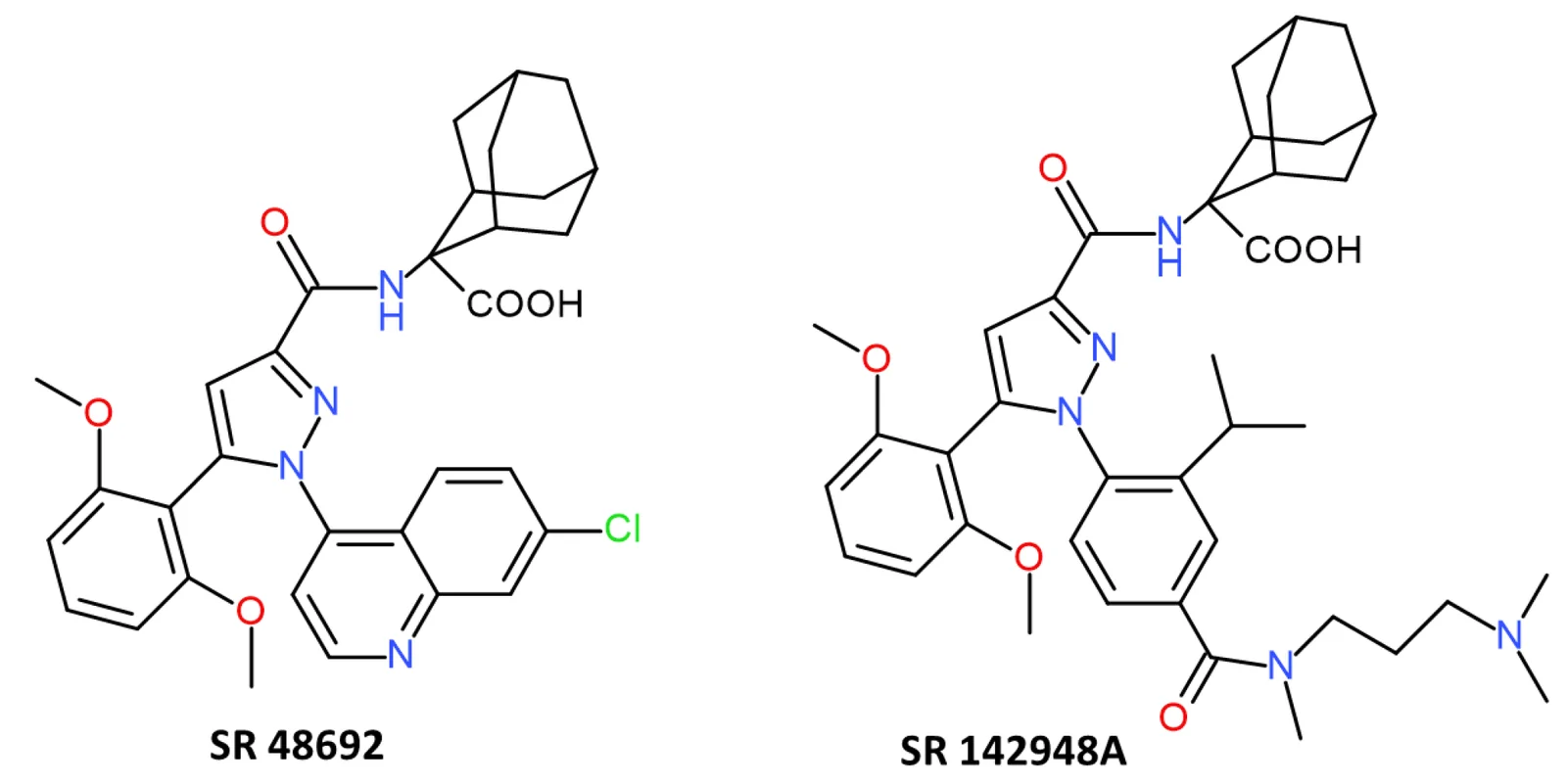
Chemical structure of the non-peptidic NTS_1_R-antagonsits. Left: SR 48692 ({2-[(1-(7-chloro-4-quinolinyl)-5-(2,6-dimethoxy-phenyl)pyrazol-3-yl)carbonylamino]tricyclo(3.3.1.1.)-decan-2-carboxylic acid}). Right: SR 142948A (2-{[5-(2,6-dimethoxyphenyl)-1-(4-(*N*-(3-dimethyl-aminopropyl)-*N*-methylcar-bamoyl)-2-isopropylphenyl)-1*H*-pyrazole-3-carbonyl]amino}ada-mantane-2-carboxylic acid).

**Figure 15:**
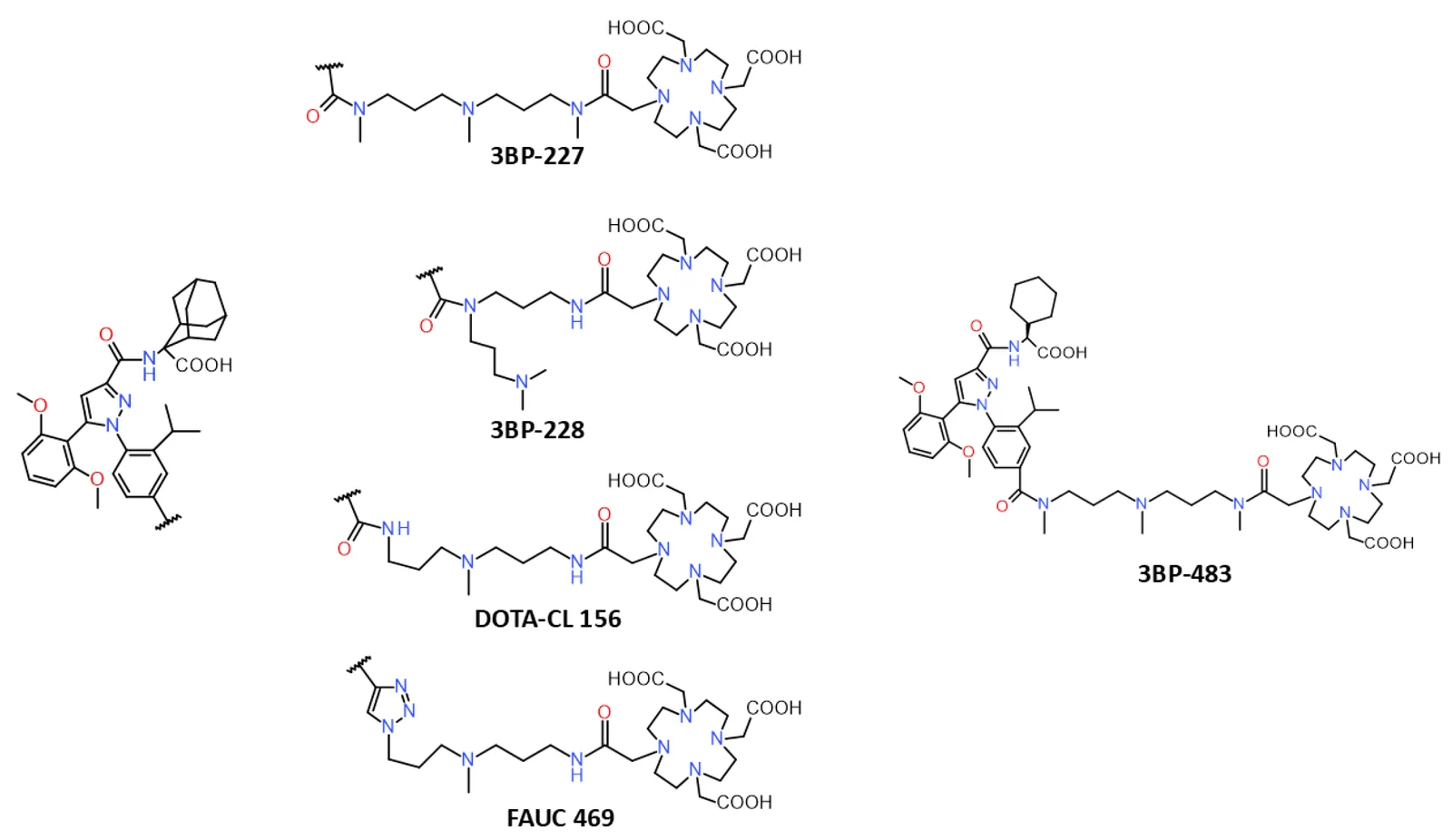
Structures of non-peptidic NTS_1_R antagonists, representing derivatized SR 142948A versions coupled to DOTA via a variety of linkers to allow for labeling with bi/trivalent radiometals used in cancer radiotheranostics. 3BP-227, 3BP-228, DOTA-CL 156 and FAUC 469 are coupled on the adamantine-containing structure depicted on the left. 3BP-483 (structure on the right) is a 3BP-227 mimic with the original adamantane group replaced by cyclohexane.

**Figure 16:**
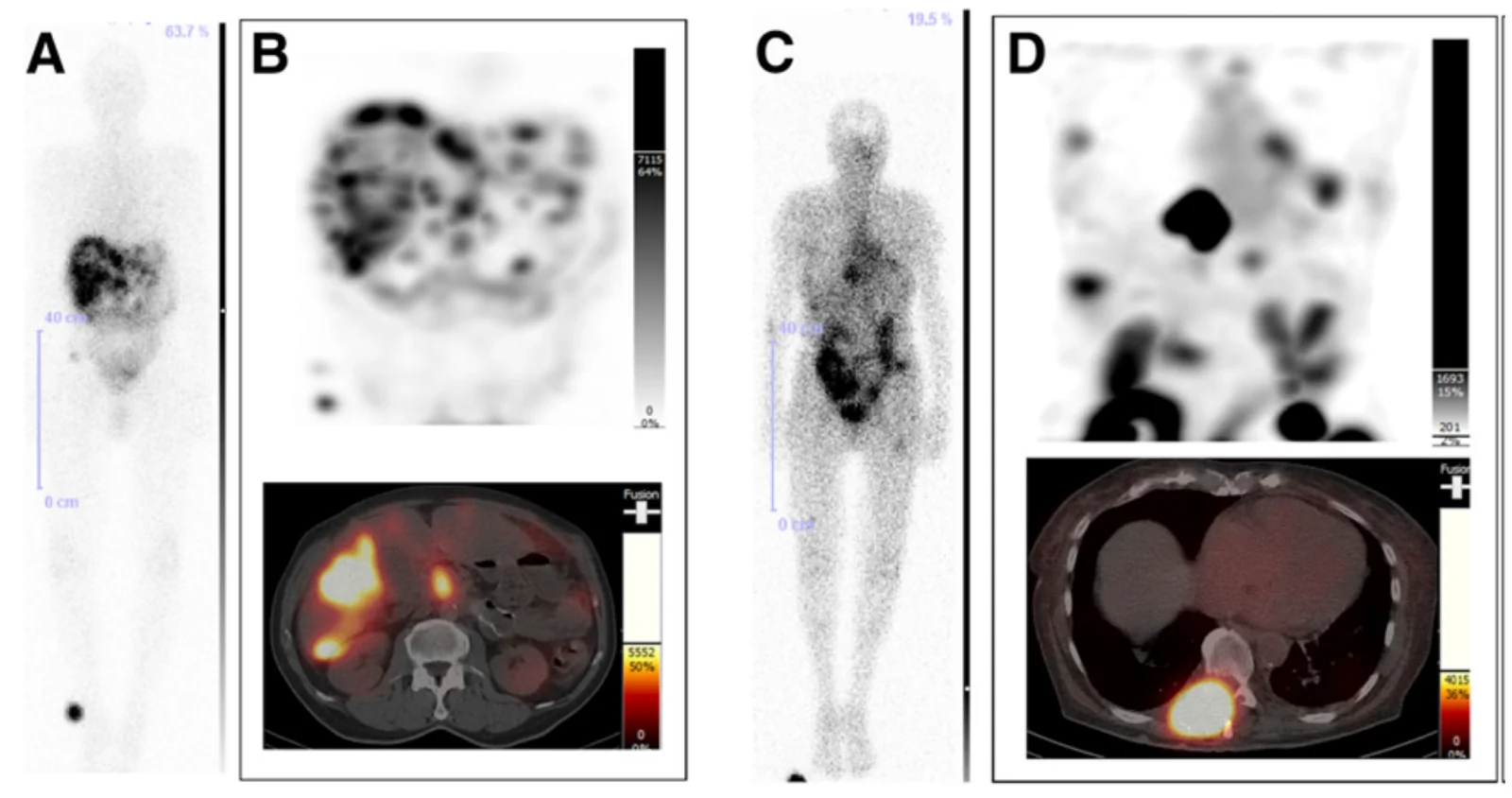
[^177^Lu]Lu-3BP-227 planar and SPECT/CT scans of PDAC patient 1 (**A**,**B**) and patient 2 (**C**,**D**). (**A**) Planar scintigraphy 24 h pi. (**B**) SPECT maximum-intensity projection 45 h pi (**top**) and axial section, liver lesions and primary tumor, 45 h pi (**bottom**). (**C**) Planar scintigraphy 48 h pi. (**D**) SPECT MIP 44 h pi (**top**) and axial section, large spinal lesion, 44 h pi (**bottom**). This research was originally published by Baum et al. in *J. Nucl. Med.* **2018** *59* (5), 809-814 [174].

**Figure 17:**
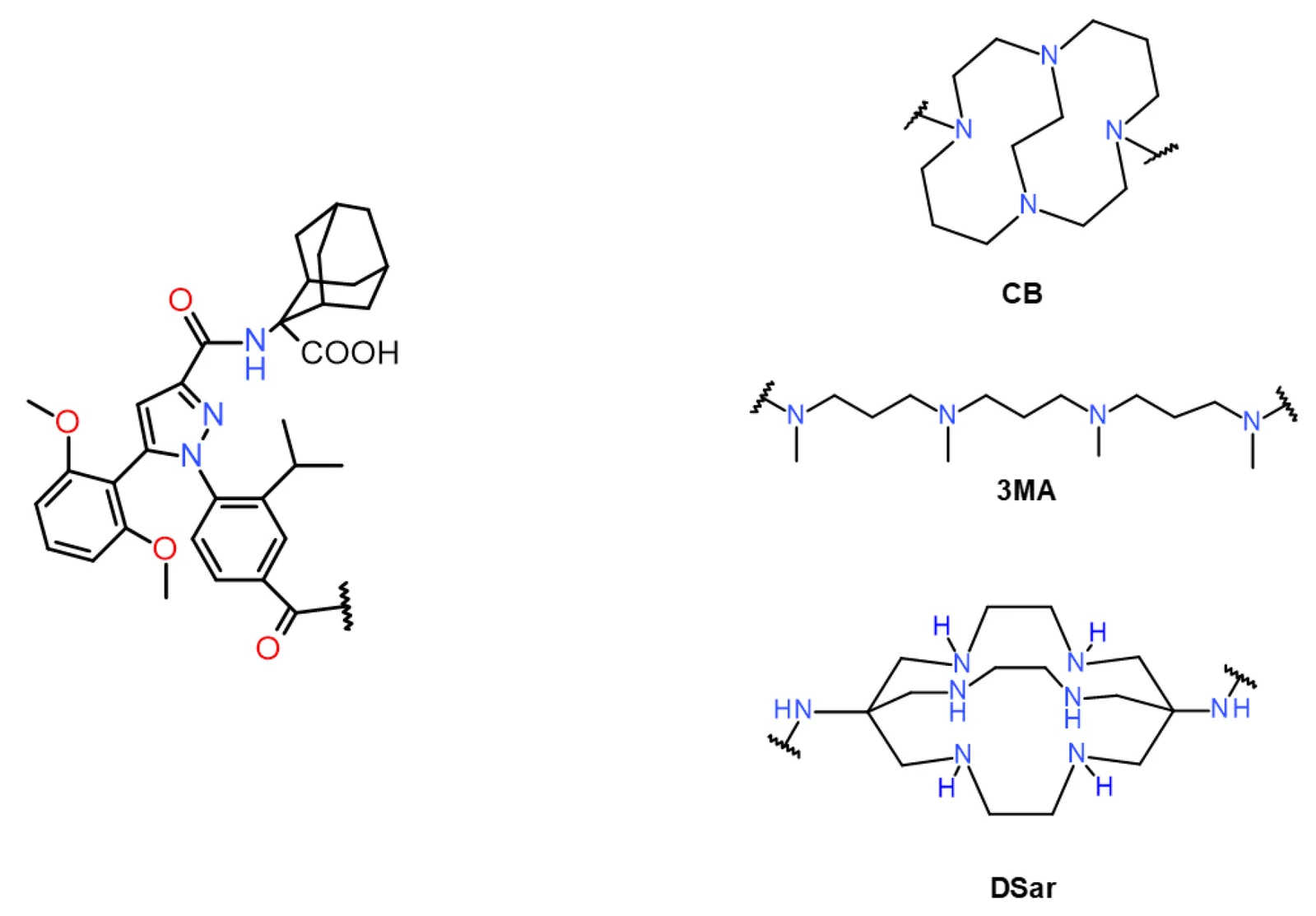
SR 142948A analogs coupled to NOTA, CB-TE2A (CB) or DSar directly or via polyamino linkers, like 3MA (MA, multi amines); alternatively, CB and DSar may serve as linkers *per se* for the attachment of the desired macrocyclic chelator (e.g., DOTA, NOTA or versions thereof).

**Table 1:** NT analogs of the NT(8–13) fragment conjugated to His or N*^a^*-Ac-His either directly or via a linker; the attached bi or tridentate co-ligand (Figure 3) enables labeling with Tc-99m or Re-186/188 via the Tc/Re-tricarbonyl methodology. Non-natural amino acids are depicted for convenience at the head of the table.

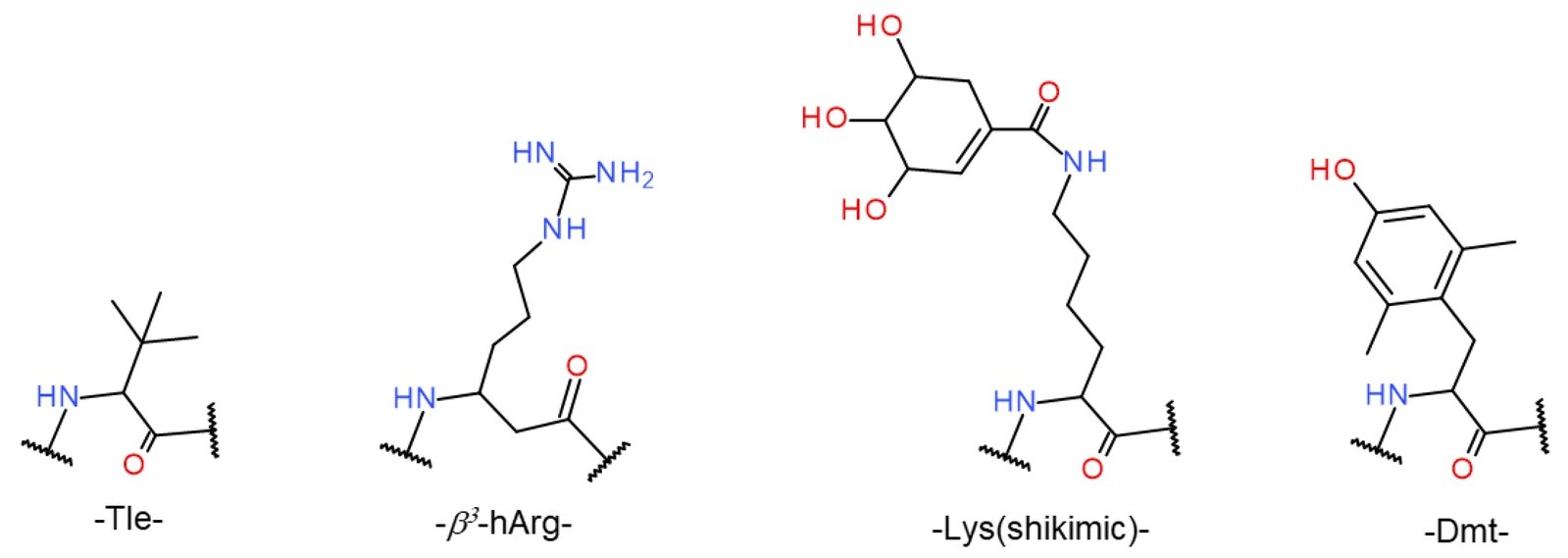				
Code Name	Co-Ligand	Linker	Sequence	References
NT(8–13)	-	-	H-Arg-Arg-Pro-Tyr-Ile-Leu-OH	
NT-I	His-	-	-Arg-Arg-Pro-Tyr-Ile-Leu-OH	[85,86]
NT-II	*N^a^*-Ac-His-	-	-Arg-Arg-Pro-Tyr-Ile-Leu-OH	[84,85,86,87,92]
NT-III	His-	-	-N(CH_3_)Arg-Lys-Pro-Tyr-Tle-Leu-OH	[86]
NT-IV	His-	-	-Lys-Ψ(CH_2_NH)-Arg-Pro-Tyr-Ile-Leu-OH	[85,86]
NT-V	*N^a^*-Ac-His-	-	-Arg-Ψ(CH_2_NH)-Arg-Pro-Tyr-Ile-Leu-OH	[86]
NT-VI	*N^a^*-Ac-His-	-	-Lys-Ψ(CH_2_NH)-Arg-Pro-Tyr-Ile-Leu-OH	[85,86,87,92]
NT-VII	*N^a^*-Ac-His-	-Asp-	-Lys-Ψ(CH_2_NH)-Arg-Pro-Tyr-Ile-Leu-OH	[86]
NT-VIII	*N^a^*-Ac-His-	-	-N(CH_3_)Arg-Lys-Pro-Tyr-Tle-Leu-OH	[88,92]
NT-IX	*N^a^*-Ac-His-	-Gly-	-Lys-Ψ(CH_2_NH)-Arg-Pro-Tyr-Tle-Leu-OH	[86]
NT-X	*N^a^*-Ac-His-	-	-Arg-Arg-Pro-Tyr-Tle-Leu-OH	[86]
NT-XI	*N^a^*-Ac-His-	-	-Lys-Ψ(CH_2_NH)-Arg-Pro-Tyr-Tle-Leu-OH	[87,93]
NT-XII	*N^a^*-Ac-His-	-	-N(CH_3_)Arg-Lys-Pro-Tyr-Tle-Leu-OH	[86,89,90,92]
NT-XIII	*N^a^*-Ac-His-	-	-*β*^3^-hArg-Arg-Pro-Tyr-Tle-Leu-OH	[90]
NT-XVIII	*N^a^*-Ac-His-	-Lys(shik)-	-Arg-(NCH_3_)Arg-Pro-Tyr-Tle-Leu-OH	[80,90]
NT-XIX	*N^a^*-Ac-His-	-	-Arg-(NCH_3_)Arg-Pro-Dmt-Tle-Leu-OH	[90,91]

Tle, *tert*-leucine; *β*^3^-hArg, beta-3 homoarginine; Lys(shik), lysine-shikimic acid; Dmt, 2,6-dimethyl-tyrosine. The structures of NT-III, NT-V, NT-VII, NT-IX and NT-X analogs could only be retrieved from a Thesis [85] and were not published in a peer-reviewed journal; the structure of NT-XIV to NT-XVII and NT-XX analogs could not be retrieved either from a publicly available Thesis document or from a peer-reviewed journal, and were thus not included in this table.

**Table 3:** Analogs of the NT(6–13) or NT(8–13) fragment conjugated to different chelators for bi/trivalent radiometals, either directly or via a linker; non-natural amino acids and linkers are depicted for convenience at the head of the table.

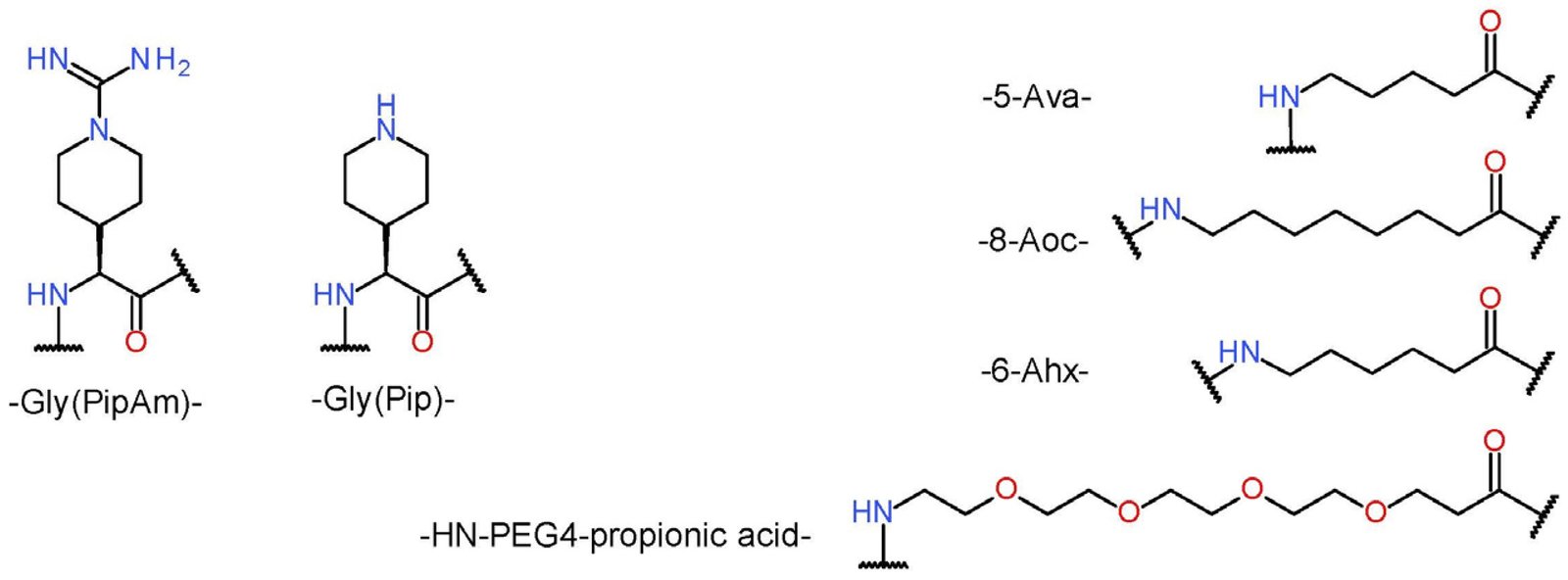				
Code Name	Chelator	Linker	Sequence	References
NT(6–13)	-	-	H-Lys-Pro-Arg-Arg-Pro-Tyr-Ile-Leu-OH	
MP-2530	DTPA-	-	-Gly(Pip)-Pro-Gly(PipAm)-Arg-Pro-Tyr-Tle-Leu-OH	[139,140,141,142,143,144]
MP-2656	DOTA-	-	-Gly(Pip)-Pro-Gly(PipAm)-Arg-Pro-Tyr-Tle-Leu-OH	[140,143]
N0	DOTA-	-	-Lys-Pro-N(CH_3_)Arg-Arg-Pro-Dmt-Tle-Leu-OH	[145]
N1	DOTA-	-*β-*Ala-	-Lys-Pro-N(CH_3_)Arg-Arg-Pro-Dmt-Tle-Leu-OH	[145]
N2	DOTA-	-5-Ava-	-Lys-Pro-N(CH_3_)Arg-Arg-Pro-Dmt-Tle-Leu-OH	[145]
N3	DOTA-	-8-Aoc	-Lys-Pro-N(CH_3_)Arg-Arg-Pro-Dmt-Tle-Leu-OH	[145]
N’1	DOTA-	-	-Arg-Arg-Pro-Tyr-Ile-Leu-OH	[146,147]
N’2	DOTA-	-PEG4*-	-Arg/Lys-Arg/Lys-Pro-Tyr-Ile/Tle-Leu-OH	[146,147]
N’3	DOTA-	-Ahx-	-Arg-Arg-Pro-Tyr-Ile-Leu-OH	[146]

Gly(Pip), 4-piperidinyl-*S*-glycine; Gly(PipAm), 4-piperidinyl(*N*-amidino)-*S*-glycine; *tert*-leucine; *t*BuGly, *tert*-butyl-*S*-glycine identical to Tle; 5-Ava, 5-aminovaleric acid, or 5-aminopentanoic acid; 8-Aoc, 8-aminooctanoic acid; PEG4*, amine-PEG4-propionic acid; Ahx, aminohexanoic acid.

**Table 5:** Silylated and non-silylated analogs of NT(8–13), conjugated to DOTA via different linkers for the binding of bi/trivalent radiometals; non-natural amino acids and linkers are depicted for convenience at the head of the table.

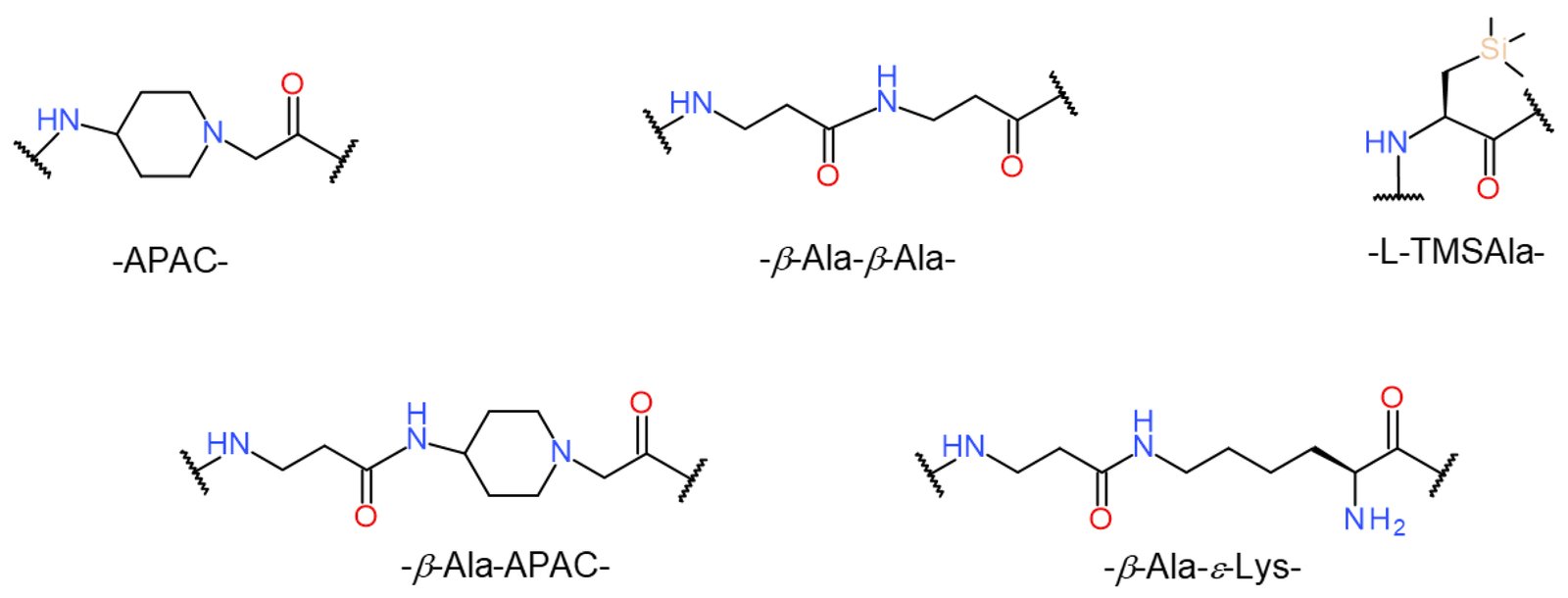				
Code Name	Chelator	Linker	Sequence	References
NT(8–13)	-	-	H-Arg-Arg-Pro-Tyr-Ile-Leu-OH	
JMV6661	DOTA-	-APAC-	-Arg-Arg-Pro-Tyr-Ile-Leu-OH	[153,154]
JMV6660	DOTA-	-APAC-	-Lys-Lys-Arg-Pro-Tyr-Ile-Leu-OH (JMV438)	[153,154]
JMV6659	DOTA-	-APAC-	-Lys-Lys-Arg-Pro-Tyr-Ile-TMSAla-OH (JMV2007)	[153,154]
JMV6658	DOTA-	-APAC-	-Lys-Lys-Arg-Pro-Tyr-TMSAla-Leu-OH (JMV2006)	[153,154]
JMV7089	DOTA-	-*β-*Ala-*β-*Ala-	-Lys-Lys-Arg-Pro-Tyr-Ile-Leu-OH (JMV438)	[155]
JMV7104	DOTA-	-*β-*Ala-APAC-	-Lys-Lys-Arg-Pro-Tyr-Ile-Leu-OH (JMV438)	[155]
JMV7222	DOTA-	-*β-*Ala-*β-*Ala-	-Lys-Lys-Arg-Pro-Tyr-Ile-TMSAla-OH (JMV2007)	[155]
JMV7124	DOTA-	-APAC-	-Lys-Ψ(CH_2_NH)-Lys-Arg-Pro-Tyr-Ile-Leu-OH	[155]
JMV7258	DOTA-	-*β-*Ala-*β-*Ala-	-Lys-Ψ(CH_2_NH)-Lys-Arg-Pro-Tyr-Ile-TMSAla-OH	[155]
JMV7259	DOTA-	-APAC-	-Lys-Ψ(CH_2_NH)-Lys-Arg-Pro-Tyr-Ile-TMSAla-OH	[155]
JMV7490	DOTA-	-*β-*Ala-*ε-*Lys-	-Lys-Ψ(CH_2_NH)-Lys-Arg-Pro-Tyr-Ile-TMSAla-OH	[155]

APAC, 2-(4-aminopiperidin-1-yl)acetic acid; TMSAla-OH, L-trimethylsilylalanine; *β-*Ala, beta-alanine; -*β-*Ala-*ε-*Lys beta-alanine-*ε-*Lys(NH_2_).

**Table 6:** NT(6–13) analogs carrying the chelator (DTPA, DOTA or NOTA: from top to bottom in the scheme at the head of the Table) coupled directly (Ahx_0_) or via a linker (Ahx_1_) at the *ε*-NH_2_ of Lys^6^; the *α*-NH_2_ of Lys^6^ has been acetylated, as shown in the Scheme at the head of the table; residues at position 11 and 12 may differ, as well as the methylation or not of Pro^7^–Arg^8^ or Arg^8^–Arg^9^ bonds. In [Lys^6^(DTPA)]-NT the DTPA has been attached at Lys^6^ of the entire NT native sequence.

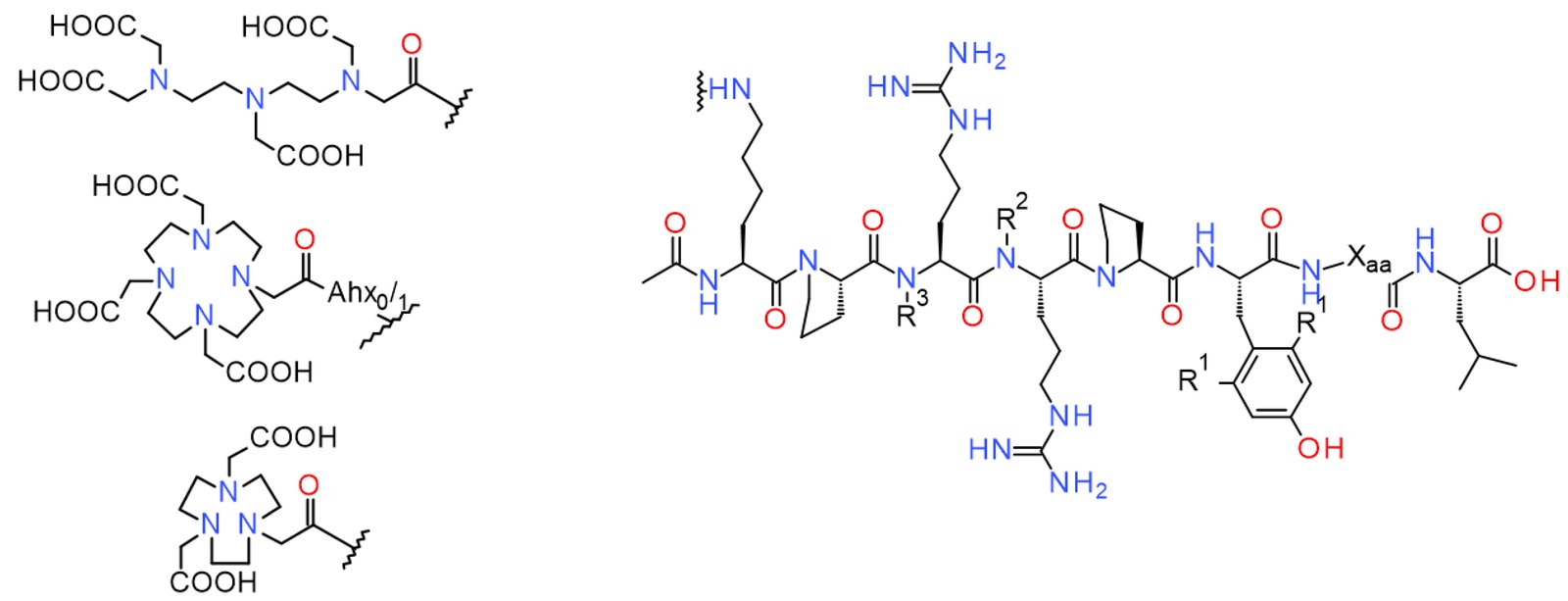				
Code Name	Chelator	Linker	Sequence	References
[Lys^6^(DTPA)]-NT	DTPA-	-	pGlu-Leu-Tyr-Glu-Asn-Lys(DTPA)-Pro-Arg-Arg-Pro-Tyr-Ile-Leu-OH	[150]
DTPA-NT-20.1	DTPA-	-	Ac-Lys(DTPA)-Pro-Arg-Arg-Pro-Tyr-Ile-Leu-OH	[150]
DTPA-NT-20.2	DTPA-	-	Ac-Lys(DTPA)-Pro-Arg-Arg-Pro-Tyr-Tle-Leu-OH	[150]
DTPA-NT-20.3	DTPA-	-	Ac-Lys(DTPA)-Pro-(NCH_3_)Arg-Arg-Pro-Tyr-Tle-Leu-OH	[150,157]
DOTA-NT-20.3	DOTA-	-	Ac-Lys(DOTA)-Pro-(NCH_3_)Arg-Arg-Pro-Tyr-Tle-Leu-OH	[157,158,159]
DOTA-NT-20.4	DOTA-	-	Ac-Lys(DOTA)-Pro-Arg-(NCH_3_)Arg-Pro-Tyr-Tle-Leu-OH	[157]
DOTA-LB119	DOTA-	Ahx- *	Ac-Lys(Ahx-DOTA)-Pro-(NCH_3_)Arg-Arg-Pro-Dmt-Tle-Leu-OH	[157]
NOTA-NT-20.3	NOTA-	-	Ac-Lys(NOTA)-Pro-(NCH_3_)Arg-Arg-Pro-Tyr-Tle-Leu-OH	[160,161]

* Ahx, aminohexanoic acid; R^2^/R^3^, H or CH_3_; R^1^, H (Tyr) or CH_3_ (Dmt); X_aa_, Ile or Tle.

## Data Availability

No new data were created or analyzed in this study. Data sharing is not applicable.
